# Removal of Misincorporated Ribonucleotides from Prokaryotic Genomes: An Unexpected Role for Nucleotide Excision Repair

**DOI:** 10.1371/journal.pgen.1003878

**Published:** 2013-11-07

**Authors:** Alexandra Vaisman, John P. McDonald, Donald Huston, Wojciech Kuban, Lili Liu, Bennett Van Houten, Roger Woodgate

**Affiliations:** 1 Laboratory of Genomic Integrity, National Institute of Child Health and Human Development, National Institutes of Health, Bethesda, Maryland, United States of America; 2 Department of Pharmacology and Chemical Biology, University of Pittsburgh Cancer Institute, Pittsburgh, Pennsylvania, United States of America; Duke University, United States of America

## Abstract

Stringent steric exclusion mechanisms limit the misincorporation of ribonucleotides by high-fidelity DNA polymerases into genomic DNA. In contrast, low-fidelity *Escherichia coli* DNA polymerase V (pol V) has relatively poor sugar discrimination and frequently misincorporates ribonucleotides. Substitution of a steric gate tyrosine residue with alanine (*umuC*_Y11A) reduces sugar selectivity further and allows pol V to readily misincorporate ribonucleotides as easily as deoxynucleotides, whilst leaving its poor base-substitution fidelity essentially unchanged. However, the mutability of cells expressing the steric gate pol V mutant is very low due to efficient repair mechanisms that are triggered by the misincorporated rNMPs. Comparison of the mutation frequency between strains expressing wild-type and mutant pol V therefore allows us to identify pathways specifically directed at ribonucleotide excision repair (RER). We previously demonstrated that rNMPs incorporated by *umuC*_Y11A are efficiently removed from DNA in a repair pathway initiated by RNase HII. Using the same approach, we show here that mismatch repair and base excision repair play minimal back-up roles in RER *in vivo*. In contrast, in the absence of functional RNase HII, *umuC*_Y11A-dependent mutagenesis increases significantly in Δ*uvrA*, *uvrB5* and Δ*uvrC* strains, suggesting that rNMPs misincorporated into DNA are actively repaired by nucleotide excision repair (NER) *in vivo*. Participation of NER in RER was confirmed by reconstituting ribonucleotide-dependent NER *in vitro*. We show that UvrABC nuclease-catalyzed incisions are readily made on DNA templates containing one, two, or five rNMPs and that the reactions are stimulated by the presence of mispaired bases. Similar to NER of DNA lesions, excision of rNMPs proceeds through dual incisions made at the 8^th^ phosphodiester bond 5′ and 4^th^–5^th^ phosphodiester bonds 3′ of the ribonucleotide. Ribonucleotides misinserted into DNA can therefore be added to the broad list of helix-distorting modifications that are substrates for NER.

## Author Summary

Most DNA polymerases differentiate between ribo- and deoxyribonucleotides quite effectively, thereby deterring insertion of nucleotides with the “wrong” sugar into chromosomes. Nevertheless, a significant number of ribonucleotides still get stably incorporated into genomic DNA. *E.coli* pol V is among the most inaccurate DNA polymerases in terms of both sugar selectivity and base substitution fidelity. The *umuC*_Y11A steric gate variant of pol V is even less discriminating when selecting sugar of the incoming nucleotide while keeping a similar capacity to form non-Watson-Crick base pairs. In the present study, we describe mechanisms employed by *E. coli* to excise rNMPs from DNA and to concomitantly reduce the extent of spontaneous mutagenesis induced by *umuC*_Y11A. The first line of defense comes from Ribonuclease HII, which initiates the ribonucleotide excision repair pathway. In the absence of RNase HII, alternate repair pathways help remove the misincorporated ribonucleotides. Here, we present the first direct evidence that nucleotide excision repair (NER) has the capacity to recognize both correctly and incorrectly paired rNMPs embedded in DNA. The combined actions of RNase HII and NER thereby reduce the mutagenic potential of ribonucleotides errantly incorporated into prokaryotic genomes.

## Introduction

In order to preserve the properties and functions of a living organism, the genetic information encoded in its DNA should be kept essentially unchanged. In reality, DNA is constantly subjected to numerous attacks from endogenous and exogenous sources changing its chemical composition and structure. If left unrepaired, these changes could have potentially serious cytotoxic and/or mutagenic consequences for the cell. Among all abnormalities in DNA, the presence of ribonucleotides in the DNA backbone appears to be one of the most common threats to genomic stability. Because of the reactive 2′-hydroxyl group on the sugar moiety, rNMPs embedded in the chromosome make the DNA strand susceptible to spontaneous and enzymatically-catalyzed hydrolytic cleavage [1]. They can also cause B- to A-form helical transition in DNA that would interfere with normal binding of various DNA-interacting proteins and disrupt a range of DNA transactions [2], [3]. Moreover, unrepaired ribonucleotides can lead to replication stress and genome instability [4]–[7].

RNA primers synthesized during the initiation of lagging-strand replication are a major source of rNMPs in DNA. These primers must be excised from DNA prior to joining of Okazaki fragments into an intact lagging strand. Several nucleases have been implicated in this process [8]. Among these are enzymes specifically hydrolyzing the phosphodiester bond between ribo- and deoxyribonucleotides, i.e. ribonucleotide-specific endonucleases, ribonucleases HI and HII (RNase H), which appeared to be ideally suited to play a primary role in RNA primer removal. However, subsequent studies revealed that the RNase H-initiated pathway is not the major mechanism leading to the removal of RNA primers (reviewed in [8]), although the enzymes are nevertheless essential for many key cellular processes requiring degradation of RNA from RNA/DNA hybrids. In particular, it has been shown that the RNase H pathway is indispensable for the removal of errant ribonucleotides randomly misinserted by DNA polymerases during replication and repair synthesis [9]–[11].

The role of *Saccharomyces cerevisiae* (*S. cerevisiae*) and *Escherichia coli* (*E. coli*) ribonucleases in ribonucleotide excision repair (RER) has been described in several recent publications [10]–[13]. In both organisms rNMP removal is primarily initiated by an RNase H type 2 enzyme; RNase H2 encoded by *rnh2* in eukaryotes and RNase HII encoded by *rnhB* in prokaryotes. Ribonucleases of this type possess a broad cleavage specificity effectively hydrolyzing phosphodiester bonds at the RNA-DNA junction on the templates containing RNA fragments, as well as isolated rNMPs embedded into double-stranded (ds) DNA. In contrast, type 1 ribonucleases, such as RNase H1 encoded by *rnh1* in eukaryotes and RNase HI encoded by *rnhA* in prokaryotes, require a tract of at least four consecutive ribonucleotides within the DNA strand for the efficient cleavage.

Biochemical analysis using yeast purified recombinant proteins revealed that RNase H1 cannot substitute for RNase H2 in the RER pathway [10]. On the other hand, using an *in vivo* approach, we have recently shown that RNase HI substitutes for RNase HII in Δ*rnhB* cells thus limiting the mutagenic consequences of excessive ribonucleotide accumulation in *E.coli* genome [11]. The apparent discrepancy between these two studies is most likely explained by differences in sugar selectivity of the polymerases responsible for rNMPs insertion, rather than by differences in substrate specificities, or other biochemical properties of yeast and bacterial type 1 ribonucleases that govern the participation of the enzymes in the RER pathway. Indeed, both yeast replicative polymerases, pol δ and pol ε effectively discriminate between rNTPs and dNTPs and incorporate ribonucleotides into DNA at low frequencies (1 per ∼600–900 nt; [10]). It is therefore highly unlikely that either pol δ or pol ε would catalyze synthesis of DNA containing several consecutive ribonucleotides, which would be a potential substrate for RNase HI. In contrast, *E. coli* pol V (UmuD′_2_C heterotrimer) appears to be one of the most indiscriminate polymerases for sugar selection [14]. In the presence of rNTPs, it is able to synthesize remarkably long RNA products [14]. A Y11A substitution in the steric gate of UmuC not only further reduces the selectivity against single rNTP incorporation, but also essentially converts the resulting mutant into a *bona fide* primer-dependent RNA polymerase that synthesizes RNA products at a 3-fold faster rate relative to the wild-type enzyme [14]. It is not surprising, therefore, that the mutant pol V catalyzes synthesis of DNA strands containing not only scattered single rNMPs, but also continuous RNA fragments that could be cleaved by both RNase HI and RNase HII [14]. Thus, while RNase HII plays a major role in keeping the *E. coli* chromosome free from errant ribonucleotides, in its absence RNase HI functions as an effective substitute to reduce genomic instability promoted through frequent ribonucleotide misincorporation.

In contrast, in the absence of a proper substitute for yeast RNase H2, replicative stress occurs and leads to genome instability [12]. This instability depends on the activity of topoisomerase 1 (Top1), whose primary function in the cell is to regulate DNA supercoiling by creating transient single-strand (ss) breaks. When Top1 cleaves the phosphodiester bond at the sites of incorporated rNMP, the ss break that is created is irreversible because of the presence of a 2′-OH group of the ribose ring. This leads to the formation of a 2′–3′-cyclic phosphate that is refractory to re-ligation [12]. Indeed, substitution of RNase H2 by Top1 in the processing of rNMPs has distinct mutagenic consequences, i.e. accumulation of 2- to 5-bp deletions within tandem repeat sequences [12].

There is no doubt that RER initiated by type 2 RNase H is the major pathway removing errant rNMPs from ds DNA. However, as with other cellular processes, it is anticipated that alternative mechanisms limiting the impact of ribonucleotides in the genome have evolved. Indeed, as noted above, that is the case when *rnhB* is inactivated and *rnhA* helps sanitize the *E.coli* genome of errantly incorporated ribonucleotides [11]. However, whilst *umuC*_Y11A-dependent mutagenesis *relative* to wild-type pol V increased from ∼7% in *rnh* ^+^ strains to ∼39% in Δ*rnhB* strains and further increased to ∼74% in the Δ*rnhB* Δ*rnhA* strain, it was still significantly lower than that promoted by wild-type pol V, despite the fact the enzyme exhibits the same low-base selectivity *in vitro* [14]. These data suggest that additional mechanisms exist which target the ribonucleotides incorporated by *umuC*_Y11A for repair and in the process misincorporated deoxyribonucleotides are also removed from the *E.coli* genome.

We have taken advantage of the pol V phenotype to investigate the contribution of mismatch repair (MMR), base excision repair (BER) and nucleotide excision repair (NER) to ribonucleotide excision repair (RER) in *E.coli*. We find no evidence for a significant role of either MMR or BER in back-up RER pathways. Somewhat surprisingly, we discovered that there was a major contribution to RER by NER *in vivo*. By using *in vitro* assays, we confirm that NER is able to recognize and excise an isolated ribonucleotide, as well as multiple rNMPs within a short RNA fragment in dsDNA *in vitro*. Efficient ribonucleotide repair in *E.coli* and most likely other prokaryotes is, therefore, achieved though the concerted actions of *rnhB*, *rnhA* and NER.

## Results

### *In vivo* system to identify repair pathways contributing to the removal of ribonucleotides misincorporated into the *E. coli* genome

**Figure 1:**
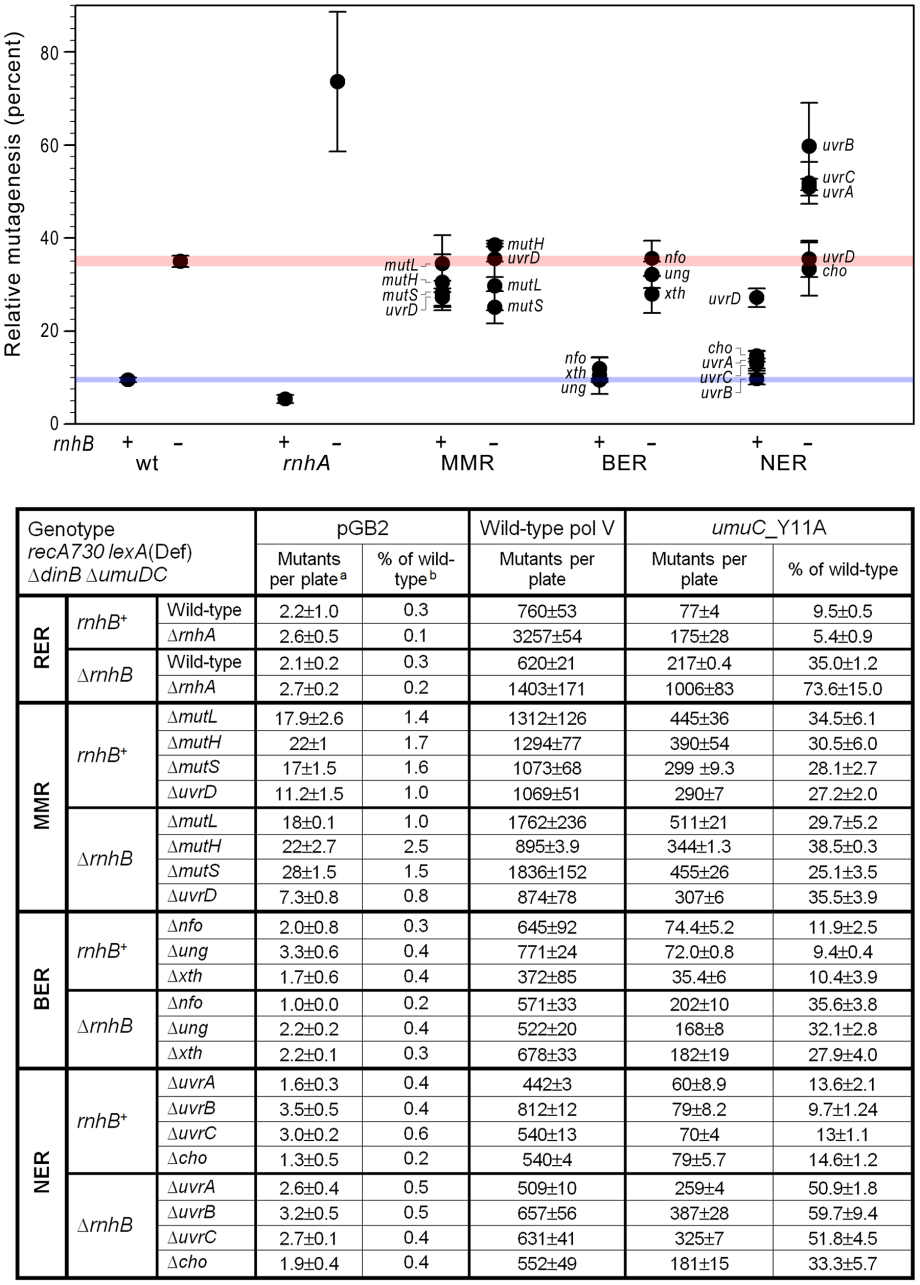
Effect of inactivating RER, MMR, BER and NER on spontaneous mutagenesis in *recA730 lexA*(Def) Δ*dinB* strains Δ*umuDC*. We constructed a series of isogenic *recA730 lexA*(Def) Δ*dinB* Δ*umuDC rnhB* ^+^/Δ*rnhB* strains in which ribonucleotide excision repair (RER; Δ*rnhA*), mismatch repair (MMR; Δ*mutL*, Δ*mutH*, Δ*mutS*, Δ*uvrD*), base excision repair (BER; Δ*ung*, Δ*xth*, Δ*nfo*), or nucleotide excision repair (NER; Δ*uvrA*, *uvrB5*, Δ*uvrC*, Δ*cho*, Δ*uvrD*) were inactivated. The extent of spontaneous mutagenesis in strains harboring the control vector, pGB2, or plasmids expressing wild-type pol V, or *umuC*_Y11A was determined by assaying reversion of the *hisG4* (ochre) allele (leading to histidine prototrophy). The average number of spontaneously arising His^+^ mutants per plate ± standard error of the mean (a) and the *relative* spontaneous mutagenesis in cells containing pGB2 vector or expressing the *umuC*_Y11A variant (b) are shown in the table below the graph. The data plotted on the graph represent the *relative* extent of spontaneous mutagenesis in cells expressing *umuC*_Y11A. The level of mutagenesis was calculated by subtracting the number of pre-existing mutants that grew on plates lacking histidine from the number of His^+^ mutants spontaneously arising on plates containing 1 µg/ml histidine. The *relative* amount of pol V-independent or *umuC*_Y11A-dependent mutagenesis was calculated as a percentage of the mutagenesis in cells expressing wild-type pol V. All experiments were performed in triplicate. Standard errors were calculated taking into account variability in spontaneous mutagenesis in each strain. The horizontal blue and red strips on the graph provide a reference baseline corresponding to the level of *umuC*_Y11A -dependent mutagenesis in repair-proficient *rnhB* ^+^ and Δ*rnhB* cells, respectively. Data for wild-type and Δ*rnhA* strains was taken from [11] and is shown for comparison. As clearly observed, inactivation or *rnhA* and NER (Δ*uvrA*, *uvrB5*, Δ*uvrC*) in a Δ*rnhB* background leads to a dramatic increase in *umuC*_Y11A-dependent mutagenesis, indicating potential roles in RER.

*E. coli* pol V is a highly error-prone Y-family DNA polymerase best characterized for its capacity to replicate damaged DNA [15], [16]. However, in certain genetic backgrounds, such as in *recA730* strains in which the RecA protein is in a so-called constitutively “activated state” (RecA*) that both favors the formation of pol V (UmuD′_2_C) [17], [18], and also increases its stability [19], [20], pol V can compete with the cell's replicase (pol III) for access to undamaged genomic DNA. Since pol V has much lower fidelity than pol III, this is manifested as a dramatic increase in spontaneous mutagenesis [21], [22]. We have previously taken advantage of this phenotype to elucidate pathways of ribonucleotide repair in *E.coli*. To do so, we generated a steric-gate Y11A mutant in the catalytic UmuC subunit of pol V, which has significantly reduced sugar discrimination. As a result, the mutant pol V enzyme incorporates ribonucleotides into DNA nearly as efficiently as deoxyribonucleotides [14]. In contrast, the base-selection fidelity of the Y11A mutant was largely unchanged and like wild-type pol V, *umuC*_Y11A frequently misincorporated the wrong base into nascent DNA *in vitro* [14]. However, to our surprise, the spontaneous mutation frequency in a *recA730 lexA*(Def) Δ*dinB* Δ*umuDC* strain expressing *umuC*_Y11A was an order of magnitude lower than that of the isogenic strain expressing wild-type pol V [23]. The apparent discrepancy was explained by the *umuC*_Y11A-dependent incorporation of rNMPs into the *E.coli* genome that triggered efficient ribonucleotide excision repair (RER) pathways and concomitantly removed misincorporated deoxyribonucleotides. *umuC*_Y11A-dependent spontaneous mutagenesis increased significantly in a Δ*rnhB* background and to a much larger extent when both *rnhB* and *rnhA* were inactivated [11] (Fig. 1). However, mutagenesis was still lower than that of wild-type pol V suggesting that other back-up RER pathways exist in *E.coli* that operate to remove errantly incorporated ribonucleotides. We hypothesized that similar to our earlier observation, where the extent of *umuC*_Y11A-dependent mutagenesis relative to wild-type pol V increased when *rnhA* was inactivated in an Δ*rnhB* strain, we would also observe an increase in *umuC*_Y11A-dependent mutagenesis in other genetic backgrounds that are compromised for RER. We therefore constructed a series of isogenic *recA730 lexA*(Def) Δ*dinB* Δ*umuDC rnhB* ^+^/Δ*rnhB* strains in which mismatch repair (MMR; Δ*mutL*, Δ*mutH*, Δ*mutS*, Δ*uvrD*), base excision repair (BER; Δ*ung*, Δ*xth*, Δ*nfo*), or nucleotide excision repair (NER; Δ*uvrA*, *uvrB5*, Δ*uvrC*, Δ*cho*, Δ*uvrD*) were inactivated and assayed for pol V-dependent spontaneous mutagenesis (Fig. 1). As noted previously, despite being isogenic, the strains expressing wild-type pol V exhibit quite different levels of spontaneous mutagenesis. We believe that this phenotype is due to effects on the constitutive activation of the RecA protein, which is an absolute requirement for high levels of pol V-dependent spontaneous mutagenesis [21]. As a consequence, we report the extent of *umuC*_Y11A-dependent mutagenesis *relative* to wild-type pol V, since any indirect effect on RecA activation would be the same for both mutant and wild-type pol V, with the only difference being their respective ability to efficiently incorporate ribonucleotides into genomic DNA. As a control, we monitored spontaneous mutagenesis in the isogenic strains lacking pol V (containing the plasmid vector, pGB2). Since the number of His^+^ revertants in these cells is pol V-independent, it should remain constantly low in all genetic backgrounds with the exception of the MMR deficient strains, where a ∼10-fold increase in mutation frequency is anticipated.

### Contribution of MMR to the removal of ribonucleotides from the *E.coli* genome

**Figure 2:**
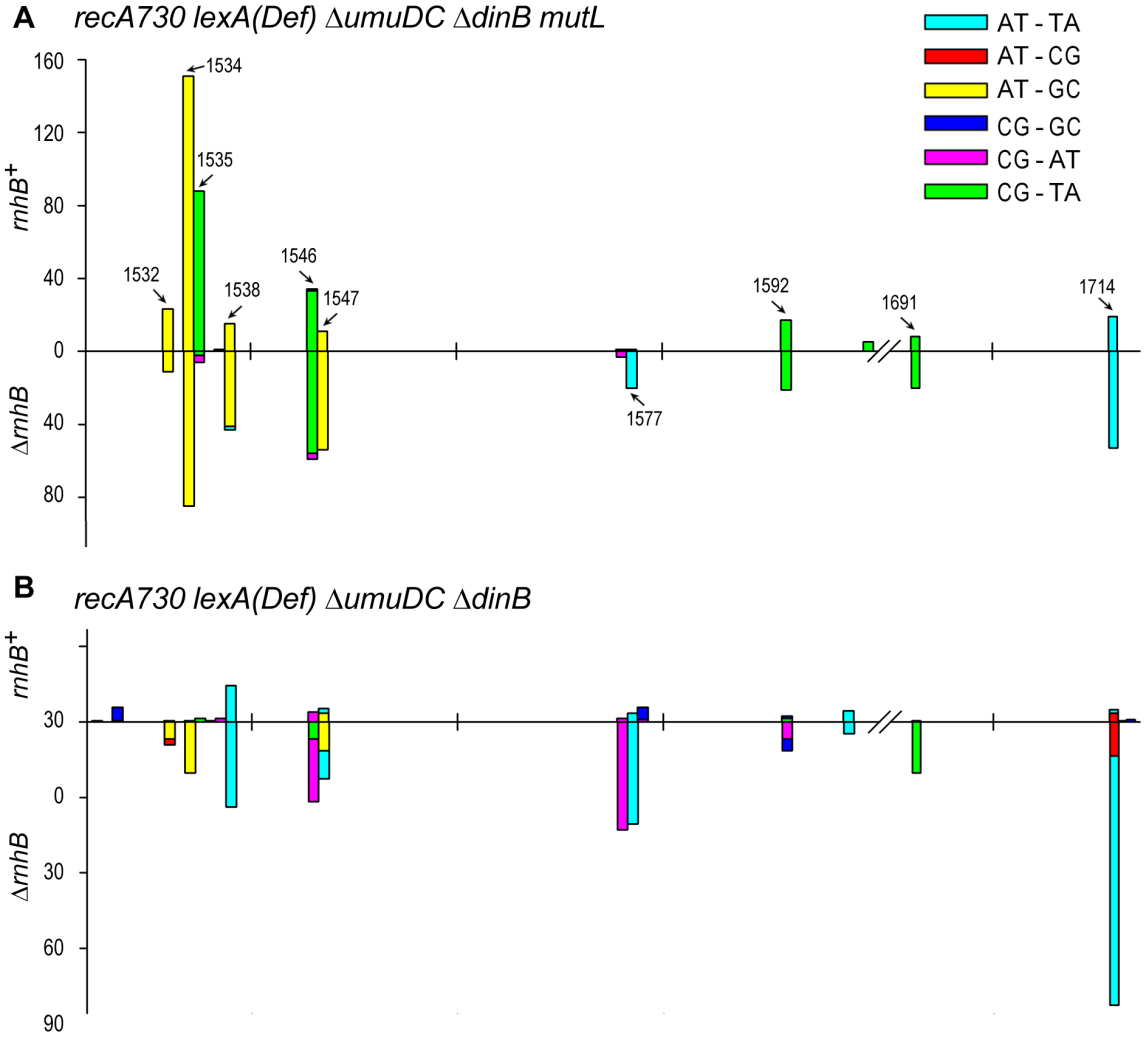
Spectra of spontaneously arising *rpoB* mutations in *recA730 lexA*(Def) Δ*umuDC* Δ*dinB* strains expressing *umuC*_Y11A and proficient- or deficient- in MMR and RNase HII- mediated RER. (**A**), Types of base-pair substitutions generated in the *rpoB* gene of mismatch repair defective *recA730 lexA*(Def) Δ*dinB* Δ*umuDC mutL rnhB*+/− strains. The arrows indicate mutagenic hot spots within *rpoB*. The spectra for the *rnhB* ^+^ strain (RW710) are taken from [14] and are shown for direct comparison with the spectra for the isogenic Δ*rnhB* strain (RW942). The forward mutation rates in the MMR^−^ strains was assayed by measuring resistance to rifampicin and were calculated as 1.22±0.2×10^−6^ for RW710 and 1.82±0.2×10^−6^ for RW942. As expected, because of defects in MMR, the spectra are dominated by transition mutations. However, we have previously reported that low-fidelity pol V makes a significant number of transversion mutations compared to other *E.coli* DNA polymerases [25] and we note a 4-fold increase in the number of transversion mutations in the Δ*rnhB mutL* strain expressing *umuC*_Y11A compared to the *mutL rnhB* ^+^ strain (see Table S1) consistent with pol V-dependent errors. The *rnhB* ^+^ *mutL* strain lacks these transversion mutations, as it undergoes active pol I-dependent RER and as a consequence, the spectrum generated in this strain actually reflects uncorrected pol I-dependent errors, rather than pol V-dependent errors. (**B**), Types of base-pair substitutions generated in the *rpoB* gene of mismatch repair proficient *recA730 lexA*(Def) Δ*dinB* Δ*umuDC rnhB*+/− strains. The forward mutation rates in the MMR^+^ strains assayed by measuring resistance to rifampicin and were calculated as 4.8±0.8×10^−8^ for RW698 and 2.5±0.4×10^−7^ for RW838. As expected, because the strains are proficient in MMR and transitions are repaired efficiently, the spectra are dominated by the poorly repaired transversion mutations. Approximately 300 Rif^R^ mutants were analyzed for each strain (Table S1). However, the spectrum of the *rnhB* ^+^ strain has been normalized to reflect the overall ∼6-fold lower mutation rate compared to the Δ*rnhB* strain. One can clearly observe that the spectra are very different in the two strains, with changes in both the types of mutations and locations of mutagenic hot-spots. Again, we believe the data support the model that in the MMR^+^ strains, pol I is responsible for low-levels of mutagenic events generated during RNase HII-pol I dependent RER, whilst in the absence of RER, *umuC*_Y11A mutations characterized by frequent transversion events persist and lead to a 6-fold increase in mutation rate.

Inactivation of MMR (via Δ*mutL*, Δ*mutH*, or Δ*mutS* alleles) results in an ∼3.5-fold increase in the relative amount of spontaneous His^+^ mutagenesis promoted by plasmid encoded *umuC*_Y11A compared to wild-type pol V (Fig. 1). These data appear to implicate MMR in the repair of at least a subset of ribonucleotides (mis)incorporated by *umuC*_Y11A and such observations are consistent with an earlier study reporting an effect of MMR on ribonucleotide repair [13]. However, there was no additional increase in the relative amount of *umuC*_Y11A mutagenesis in Δ*rnhB* Δ*mutL*, Δ*rnhB* Δ*mutH*, or Δ*rnhB* Δ*mutS* strains (Fig. 1), which would be expected if MMR participates in repair pathways that substitute for the RNase HII-dependent RER of ribonucleotides misincorporated by *umuC*_Y11A. We hypothesize that the increase in relative mutagenesis in the *rnhB* ^+^ MMR-deficient strains expressing *umuC*_Y11A is not caused by a reduction of rNMP repair, but rather reflects misincorporations made by a different DNA polymerase participating in the re-synthesis step of ribonucleotide repair. To examine this possibility, we determined the spectra of mutations generated in *rnhB* ^+^/Δ*rnhB* strains expressing *umuC*_Y11A (Fig. 2). As expected, the Δ*mutL rnhB* ^+^ spectrum (Fig. 2A) was dominated by transition events. In contrast, the Δ*mutL* Δ*rnhB* strain exhibited a different mutagenic spectrum that included many more transversions (Table S1), which are “signatures” of error-prone pol V [24], [25].

To further extend our hypothesis that the mutations in the *rnhB* ^+^ and Δ*rnhB* strains are generated by two different polymerases operating in two different pathways, we assayed the spectrum of *rpoB* mutations in mismatch repair proficient *rnhB* ^+^ and Δ*rnhB* strains expressing *umuC*_Y11A (Fig. 2B). In contrast to the MMR^−^ strains, where the spectrum was dominated by transitions, the majority of base substitutions in the MMR^+^ strains were transversion events, which is consistent with the efficient repair of transition mutations by the mismatch repair machinery (Table S1). In agreement with our hypothesis, not only was the mutation rate of the *rnhB* ^+^ strain 6-fold lower compared to the Δ*rnhB* strain, the mutagenic hot-spots varied considerably, suggesting that the mutations were generated by different DNA polymerases. In particular, in the Δ*rnhB* strain where *umuC*_Y11A misincorporations are likely to persist, the spectrum was dominated by AT→TA transversions (at positions 1547, 1577 and 1714) that are characteristic of pol V [24].

Together, our data indicate that the prokaryotic MMR pathway, while possessing the capacity to recognize mispaired bases, does not selectively recognize ribonucleotide mispairs over deoxyribonucleotides mispairs and therefore does not contribute significantly to RER.

### Contribution of BER to the removal of ribonucleotides from the *E.coli* genome

To continue our search of a RER backup pathway, we turned to base excision repair (BER), which targets a variety of base lesions. Of particular relevance, are dUMPs frequently misincorporated by DNA polymerases [26], or formed through the spontaneous hydrolytic deamination of cytosines and which are released through BER initiated by uracil DNA glycosylase (encoded by the *ung* gene). Ung recognizes the lesion and hydrolyzes the N-glycosylic bond between the uracil base and sugar ring converting uracil into an abasic site. The next major step of BER is cleavage of the abasic site by one of the class II apurinic/apyrimidinic (AP) endonucleases, such as exonuclease III (encoded by the *xthA* gene), or endonuclease IV (encoded by *nfo*), which together account for the vast majority of AP endonuclease activity in *E.coli* [27], [28]. Similar to the proposed RNase HII-dependent RER pathway, processing of the BER intermediates involves strand-displacement DNA synthesis with replication products ranging from just one base-pair to several hundred nucleotides [29]–[31]. We therefore considered the possibility that BER might operate to remove misincorporated rUMPs, which would provide a mechanism to reduce the mutagenic potential of *umuC*_Y11A-dependent spontaneous mutagenesis. However, inactivation of *ung*, *xth*, or *nfo* had no discernible effect on the relative extent of *umuC*_Y11A mutagenesis in either *rnhB* ^+^ or Δ*rnhB* strains (Fig. 1). Based upon these observations, we conclude that BER is unlikely to participate in any RER back-up pathway in *E.coli*.

### Contribution of NER to the removal of ribonucleotides from the *E.coli* genome

We recently reported that Δ*uvrA* and Δ*uvrC* strains expressing *umuC*_Y11A are as UV-resistant as those expressing wild-type pol V. This is in dramatic contrast to *uvr* ^+^ strains in which *umuC*_Y11A confers minimal UV-resistance [23] despite being as proficient as wild-type pol V in its ability to traverse a UV-induced cyclobutane pyrimidine dimer (CPD) [23]. Such phenotypes were attributed to the dual actions of RNase HII nicking the nascent TLS strand and the concomitant actions of the NER proteins on the opposite CPD-containing strand to generate lethal double-strand breaks [11]. In the present study, we analyzed the effect of NER on RER of undamaged DNA.

To do so, we determined spontaneous mutagenesis in strains carrying mutations in genes encoding key proteins that mediate damage recognition and excision steps of the *E. coli* NER pathway (*uvrA*, *uvrB*, *uvrC*, *cho* and *uvrD*). It should be noted that the *uvrA, uvrB, cho* and *uvrD* genes are normally regulated at the transcriptional level by the LexA repressor [32]. However, since the strains used for the mutagenesis assays carry the *recA730 lexA*(Def) alleles which lead to derepression of all genes in the LexA-regulon, the UvrA, UvrB, Cho and UvrD proteins are all expected to be expressed at fully derepressed levels and as a consequence, NER is active in the absence of exogenous DNA damage. Despite this fact, inactivation of *uvrA*, *uvrB*, *cho* or *uvrC* (which is not under *lexA* control), in the *rnhB* ^+^ background had little effect on the overall low level of mutagenesis promoted by *umuC*_Y11A relative to wild-type pol V (Fig. 1). The Δ*uvrD* strain exhibited somewhat higher levels of spontaneous mutagenesis. However, this phenotype is probably unrelated to NER, but is instead more in line with its dual functions in MMR [33], [34]. Interestingly, there was a significant increase in the extent of *umuC*_Y11A-dependent spontaneous mutagenesis in the Δ*uvrA* Δ*rnhB*, *uvrB5* Δ*rnhB* and Δ*uvrC* Δ*rnhB* strains (Fig. 1). In contrast, the relative extent of spontaneous mutagenesis remained essentially unchanged in the Δ*cho* Δ*rnhB*, or Δ*uvrD* Δ*rnhB* strains. Together, these observations imply that NER is able to remove ribonucleotides from DNA, and this process occurs via the “classical” NER pathway mediated by the UvrABC proteins and not through an alternate UvrAB/Cho-dependent pathway [35]. In addition, the lack of an apparent *uvrD* phenotype in RER is consistent with a previous study showing that UvrD is not necessary for the lesion removal under SOS conditions [36].

**Figure 3:**
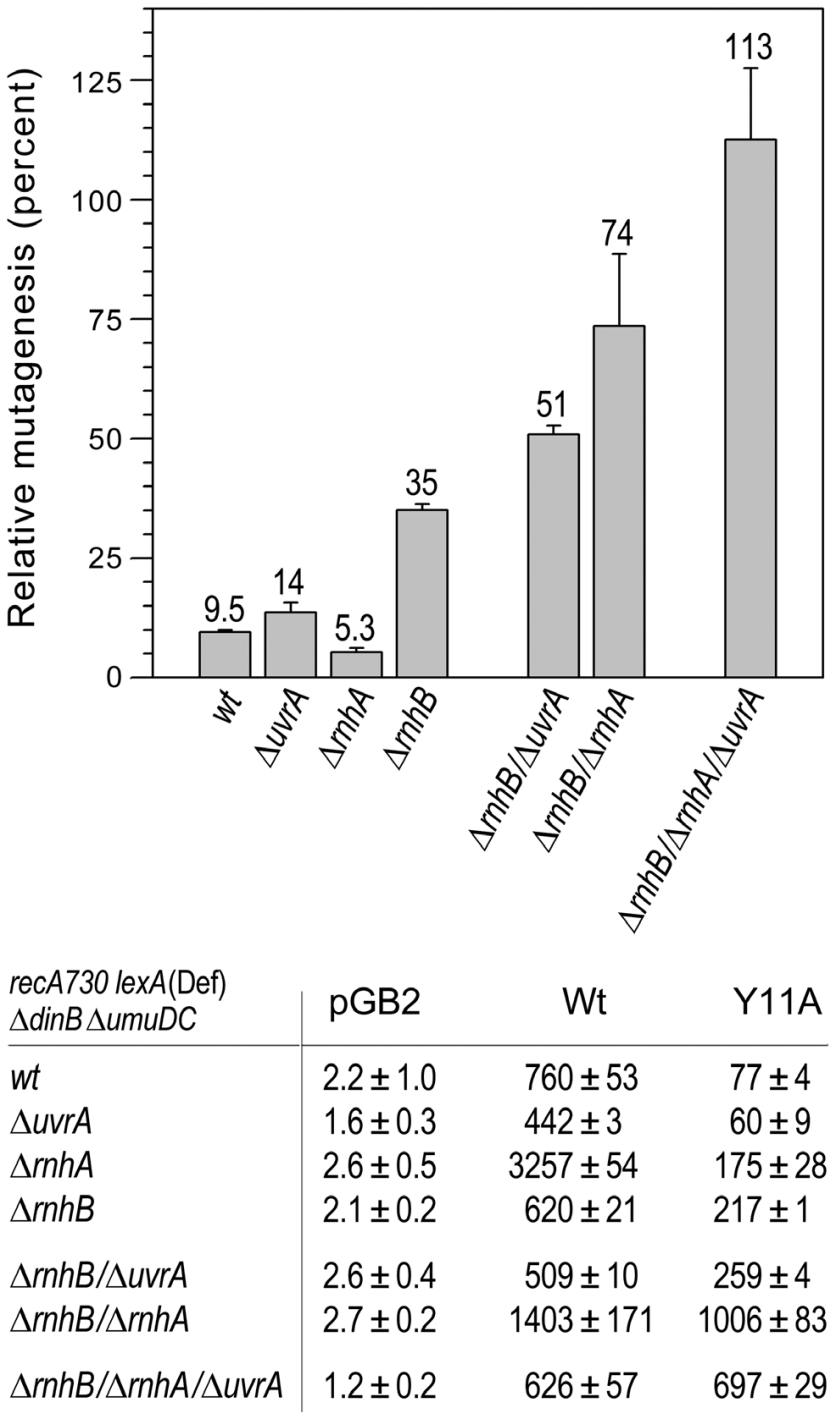
Effect of deleting *rnhA*, *rnhB* and/or *uvrA* alone, or in various combinations, on the extent of *umuC*_Y11A-dependent spontaneous mutagenesis in *recA730 lexA*(Def) Δ*umuDC* Δ*dinB* strains. We constructed a series of isogenic *recA730 lexA*(Def) Δ*dinB* Δ*umuDC* strains with Δ*rnhA*, Δ*rnhB* or Δ*uvrA* alleles alone, or in various combinations and assayed pol V-dependent spontaneous mutagenesis in strains harboring plasmids expressing wild-type pol V or *umuC*_Y11A by assaying reversion of the *hisG4* (ochre) allele. The data plotted on the graph represent the *relative* extent of spontaneous mutagenesis in cells expressing *umuC*_Y11A calculated as a percentage of the spontaneous mutagenesis in cells expressing wild-type pol V. All experiments were performed in triplicate. Standard errors were calculated taking into account variability in spontaneous mutagenesis in each strain. Data for the wild-type, Δ*rnhA*, Δ*rnhB and the* Δ*rnhA* Δ*rnhB* strains were taken from [11] and are shown for comparison. As clearly observed, in contrast to the Δ*rnhA or* Δ*uvrA* strains, which exhibit roughly the same extent of *umuC*_Y11A-dependent mutagenesis as the wild-type strain, the Δ*rnhB* strain exhibited significantly higher levels of spontaneous mutagenesis, suggesting that the *rnhB*-encoded RNase HII repair pathway is the primary defense against errant ribonucleotide incorporation in *E.coli*. However, in contrast to the *rnhB* ^+^ strains, deletion of either *rnhA* or *uvrA* in the Δ*rnhB* strain background leads to a further increase in *umuC*_Y11A-dependent spontaneous mutagenesis, suggesting that both enzymes participate in back-up pathways of ribonucleotide repair. Furthermore, *umuC*_Y11A-dependent spontaneous mutagenesis in the Δ*rnhA* Δ*rnhB* Δ*uvrA* triple mutant strain is actually higher than in the isogenic strain expressing wild-type pol V. These data imply that all major pathways specific for ribonucleotide repair are blocked in this strain background.

Furthermore, inactivation of NER (by Δ*uvrA*) in the Δ*rnhB* Δ*rnhA* strain resulted in a dramatic increase in *umuC*_Y11A-dependent mutagenesis, such that it actually became greater than that produced by wild-type pol V (Fig. 3), indicating that RER is completely inactivated in this genetic background. These findings confirm that UvrABC-dependent NER serves as a *bona fide* back-up to RNase HII-mediated RER.

In summary, our *in vivo* studies do not indicate a significant role for MMR or BER in ribonucleotide repair in *E.coli*. In contrast, inactivation of RER via mutations in *rnhB* resulted in an increase in *umuC*_Y11A-dependent mutagenesis in Δ*rnhA* and NER-deficient (Δ*uvrA*, *uvrB5* and Δ*uvrC*) strains, suggesting that RNase HI and the UvrABC proteins function in alternative ribonucleotide repair pathways in *E.coli* (Fig. 1).

### *In vitro* reconstitution of NER targeted to rNMPs embedded in a dsDNA substrate

To our knowledge, our data present the first biological evidence for the participation of prokaryotic NER proteins in ribonucleotide repair. We therefore wanted to test the ability of the UvrABC complex to remove rNMPs from DNA directly. To do so, we performed an *in vitro* incision assay to determine whether the reconstituted NER complex has the capacity to remove ribonucleotides from double-stranded (ds) DNA. For these experiments, we chose to utilize highly purified *Bacillus caldotenax* UvrA and UvrB and *Thermatoga maritima* UvrC proteins. The UvrABC proteins from the thermophilic bacteria while having extremely high level of sequence similarity with the *E. coli* proteins are remarkably more stable [37]–[41]. Furthermore, previous studies have demonstrated that individual subunits of the NER complex from Gram-positive bacteria are able to efficiently substitute for the components of the *E. coli* nuclease in various *in vitro* excision reactions [37]–[39], [42]–[46]. We have shown earlier that the UmuC_Y11A polymerase readily extends primers by very efficient (mis)incorporation of ATP opposite a variety of different template bases [14]. Therefore, the double-stranded DNA oligonucleotide used as a substrate in the *in vitro* assays contained either a single, or two consecutive rAMPs. In addition, since UmuC_Y11A is also known to replicate DNA with very low base-substitution fidelity producing both transitions and transversions, in some of the substrates either one (3′), or both rAMPs, were mispaired with either cytosine or adenine on the complementary DNA strand. The UmuC_Y11A enzyme is also characterized by an extraordinary ability to synthesize long RNA strands within minutes of engaging the primer-terminus [14]. As a consequence, we also wanted to examine whether the UvrABC proteins are able to initiate repair of DNA containing multiple ribonucleotides and for this purpose, we utilized oligonucleotides with five sequential rNMPs.

**Figure 4:**
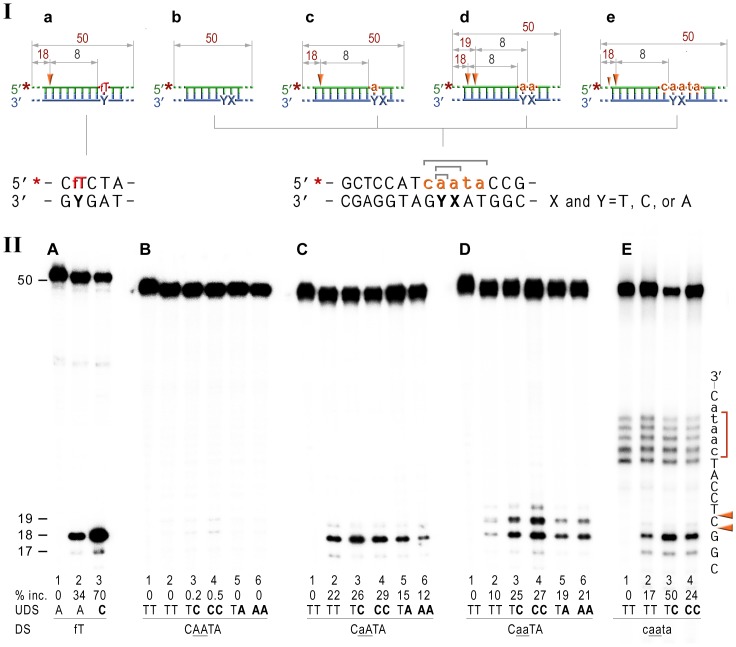
NER cleavage reaction products generated using various DNA-RNA-DNA hybrid substrates. **I**; Cartoon of the synthetic substrates used in the *in vitro* assays, with the sites of incision and expected product size indicated, along with the DNA sequence containing rNMP(s) and mismatched nucleotides. **II**; The 50-mer duplexes (10 nM) in which the modified strand was 5′ end-labeled (indicated by *), were incubated with the NER proteins at concentrations of 40 nM (UvrA), 200 nM (UvrB), and 100 nM (UvrC) for 60 min at 55°C in the presence of 1 mM ATP. UvrABC-dependent incision on the fluorescein adducted 50-bp duplex (fT) was used as a control NER activity of the purified proteins (panel A). The DNA duplexes (panel B) as well as DNA-RNA-DNA hybrids either containing a single rAMP (panels C), two consecutive rAMPs (panel D), or five rNMPs (panels E) were assayed. The reaction products were separated under denaturing conditions by 15% polyacrylamide gel electrophoresis (PAGE). The efficiency of UvrABC-dependent incision was determined as a percentage of the radioactivity in the incised products relative to the total signal of the substrate (% inc.). The data below the gels are mean values calculated from at least two independent experiments. The DNA sequence containing rNMP(s) is shown alongside the gels where DNA and RNA are represented by uppercase and lowercase letters, respectively. Orange arrows indicate the cleavage sites. The red bracket indicates spontaneous ribonucleotide cleavage. UDS, refers to the undamaged strand, and DS, is damage/ribonucleotide-containing strand. The *in vitro* assays reveal that the NER proteins incise the DNA backbone 8 base pairs 5′ of ribonucleotides and that the reactions are stimulated by base mispairs.

In the current study, we used double-stranded 50-mer oligonucleotides with rNMPs embedded into a 5′ or 3′ end-labeled DNA strand, which allows us to monitor incisions at both sides of the modified base(s) (Fig. 4). Furthermore, the duplex oligonucleotides were modeled upon a nearly identical fluorescein adducted substrate, which has previously been shown to be a good substrate for the UvrABC endonuclease *in vitro* [39] (Fig. 4A, lanes 2,3) thereby allowing us to directly compare the efficiency of lesion-mediated incision to ribonucleotide-mediated incision.

As expected, the undistorted templates remained intact (Fig. 4B, lane 2), while reactions with duplexes containing A:C and AA:CC mispairs yielded barely detectible bands corresponding to the incisions made at the 8^th^ bond 5′ to the mispair (Fig. 4B, lanes 3,4). In contrast, A:A and AA:AA mismatches did not attract UvrABC mediated incisions (Fig. 4B, lanes 5,6). Our finding is in good agreement with previously published reports of low levels (0.03–0.5%) of mismatch repair by the bacterial and human NER proteins [47], [48]. Consistent with our *in vivo* observations for a role of NER in RER, incubation of the 5′-labeled rAMP-containing DNA with UvrA, UvrB, and UvrC proteins resulted in the robust oligonucleotide cleavage (Fig. 4C, lane 2), which was only minimally less efficient than cleavage of the DNA with a single fluorescein-adducted thymine (c.f. Fig. 4A vs. 4C, lane 2). As with the lesions-containing substrate, incision of the ribonucleotide-containing substrate was made at the 8^th^ phosphodiester bond 5′ to the RNA–DNA junction producing an 18 bp product. Reactions with oligonucleotides having two sequential AMPs used as substrates for the UvrABC endonuclease yielded two bands, 18- and 19-mers (Fig. 4D, lane 2) that correspond to incisions made at the 8^th^ bond on the 5′ side of each ribonucleotide. Incision of the substrates with five rNMPs in a row mainly generated an 18 nucleotide fragment (corresponding to an incision 7 bases 5′ to the first rAMP), although a small amount of 17-mer, which is produced by the incisions of the 8^th^ bond 5′ to the RNA/DNA junction, was also observed (Fig. 4E, lane 2). In contrast and as expected, no cleavage was observed when the complementary DNA strand lacking rNMPs was labeled (Fig. S1).

**Figure 5:**
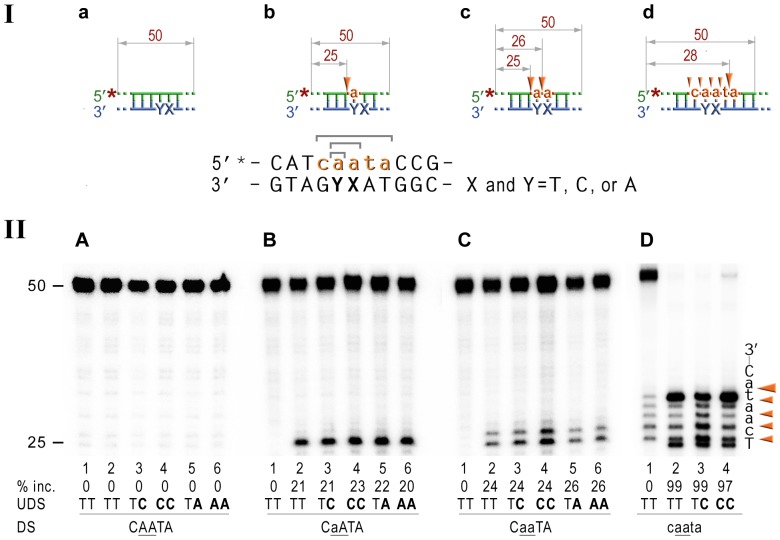
RNase HII cleavage reaction products generated using various DNA-RNA-DNA hybrid substrates. **I**; Cartoon of the synthetic substrates used in the *in vitro* assays, with the sites of incision and expected product size indicated, along with the DNA sequence containing rNMP(s) and mismatched nucleotides. **II**; The 50-mer duplexes (10 nM) in which the modified strand was 5′ end-labeled (indicated by *), were incubated with Rnase HII for 60 min at 37°C. The DNA duplexes (panel A) as well as DNA-RNA-DNA hybrids containing either single rAMP (panel B), two consecutive rAMPs (panel C), or five rNMPs (panel D) were assayed. The reaction products were separated under denaturing conditions by 15% polyacrylamide gel electrophoresis (PAGE). The efficiency of RNase HII-dependent incision was determined as a percentage of the radioactivity in the incised products relative to the total signal of the substrate (% inc.). The data below the gels are mean values calculated from at least two independent experiments. The DNA sequence containing rNMP(s) is shown alongside the gels where DNA and RNA are represented by uppercase and lowercase letters, respectively. Orange arrows indicate the cleavage sites. The *in vitro* assays confirm that *E.coli* RNase HII nicks the DNA backbone 5′ of ribonucleotides embedded in DNA and shows that the efficiency of the reaction is largely unaffected by base-mispairs.

It has been reported that NER prefers compound lesions consisting of a base damage placed opposite one or more mispaired bases, over the correctly paired lesions (Fig. 4A, lane 3) [49]–[51]. Similarly, the efficiency with which the rNMP-containing template was incised by the UvrABC endonuclease, was also determined by the type of the mispaired base (Fig. 4, B–E). Mispairing of rA with dC potentiated incision of the substrates containing one, two, or five ribonucleotides (lane 3 on the Fig. 4C, D, and E). In contrast, formation of a dA:rA mispair inhibited removal of the nucleotide with an incorrect sugar (lane 5 on the Fig. 3B, C, and D). The number of mismatches did not affect the incision efficiency for the substrates with one or two rAMPs (lanes 4 and 6 in Fig. 4C and D), but in case of the longer ribonucleotide fragment, the presence of an additional mispair (Fig. 4E, lane 4) counteracted the stimulatory effect of a single mismatch (lane 3). Among all DNA/RNA hybrids tested, the greatest incision by UvrABC was observed on the substrate with a tract of five rNMPs and one C:rA mispair (Fig. 4E, lane 3). In contrast to the UvrABC reactions, RNase HII-catalyzed cleavage 5′ to the rAMP was not stimulated by a mispaired base, but was identical for all the substrates with one or two ribonucleotides embedded into ds DNA (Fig. 5).

**Figure 6:**
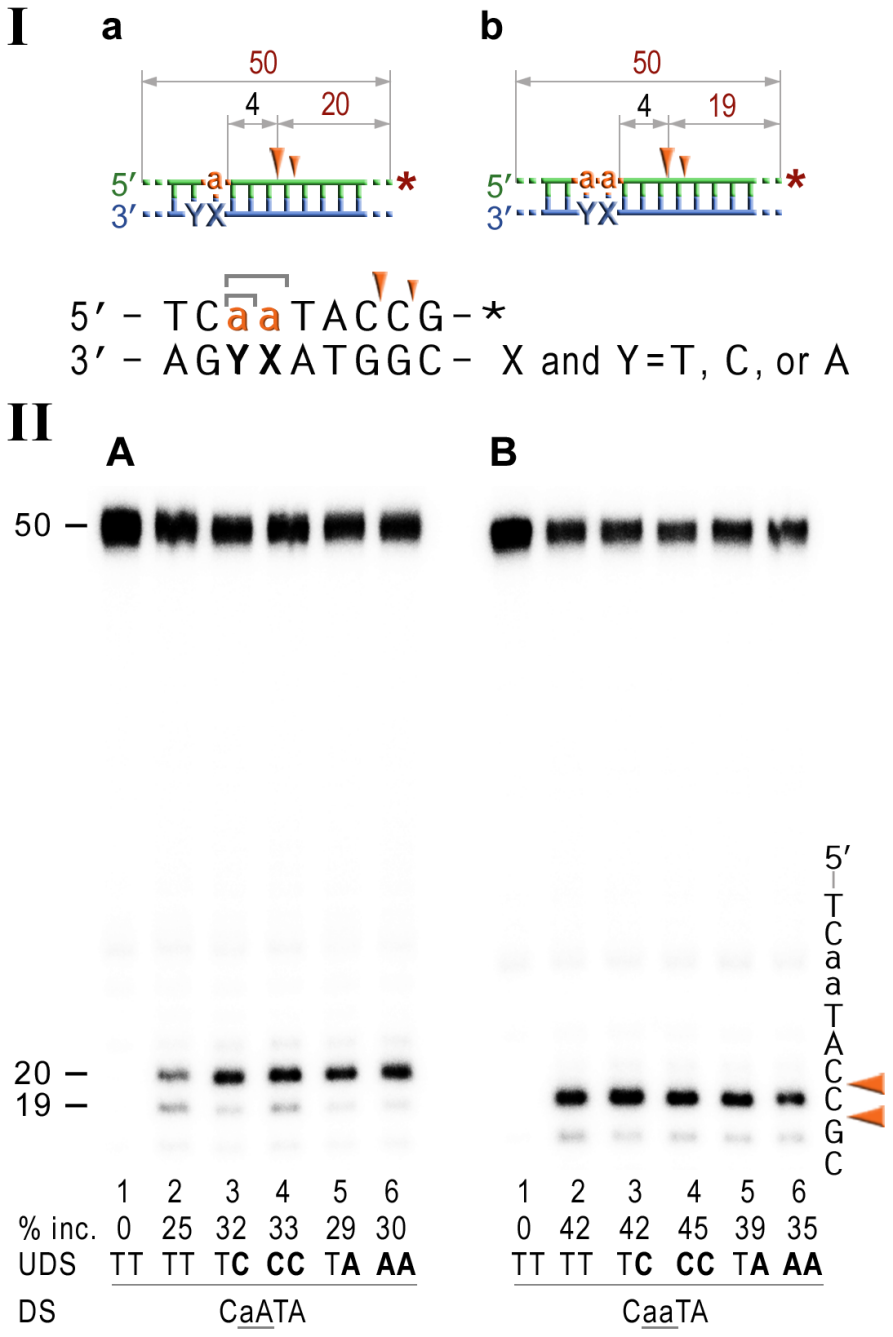
NER cleavage reaction products generated using DNA templates with 3′ end-labeled rNMP-containing strands. **I**; Cartoon of the synthetic substrates used in the *in vitro* assays, with the sites of incision and expected product size indicated, along with the DNA sequence containing rNMP(s) and mismatched nucleotides. **II**; The 50-mer duplexes (10 nM) in which the modified strand was 3′ end-labeled (indicated by *), were incubated with the NER proteins at concentrations of 40 nM (UvrA), 200 nM (UvrB), and 100 nM (UvrC) for 60 min at 55°C in the presence of 1 mM ATP. The DNA duplexes DNA-RNA-DNA hybrids either containing a single rAMP (panel A), two consecutive rAMPs (panel B) were assayed. The reaction products were separated under denaturing conditions by 15% polyacrylamide gel electrophoresis (PAGE). The efficiency of UvrABC-dependent incision was determined as a percentage of the radioactivity in the incised (% inc.) products relative to the total signal of the substrate. The data below the gels are mean values calculated from at least two independent experiments. The DNA sequence containing rNMP(s) is shown alongside the gels where DNA and RNA are represented by uppercase and lowercase letters, respectively. The full-sized DNA template (50-mer) and incision products of 19–20 bp are indicated. Orange arrows indicate the cleavage sites. The *in vitro* assays reveal that the NER proteins incise the DNA backbone 4–5 base pairs 3′ of ribonucleotides and that the efficiency of the reaction is largely unaffected by base-mispairs.

Analysis of the NER reactions using templates with 3′-labeled rNMP-containing strands indicated that the incision was made at the 5^th^ phosphodiester bond 3′ to the RNA–DNA junction producing a 20 bp (Fig. 6A), or a 19 bp (Fig. 6B) product. In contrast to the 5′ incision activity, the efficiency of the cleavage 3′ to the rNMP(s) was mainly independent of the presence of base mismatches and type and number of mispaired bases, although templates with the rAs were cleaved somewhat more efficiently than templates containing a single rAMP (Fig. 6).

## Discussion

*E. coli* pol V belongs to the Y-family of DNA polymerases [52], most of which are involved in replication of damaged or distorted DNA [53]. In order to accommodate abnormal nucleotides, the active site of these polymerases is spacious and solvent-exposed [54]. As a consequence, the polymerases exhibit low-fidelity when replicating undamaged DNA and their up-regulation in cells often confers a mutator phenotype [21], [55], [56]. Pol V differs from other Y-family members in that along with low base-substitution fidelity, it is also characterized by exceptionally low sugar selectivity [14].

Even though the majority of DNA polymerases discriminate against nucleotides with a ribose moiety quite efficiently, rNMPs appear to be among the most abundant abnormalities in chromosomal DNA [5], [57]. Nevertheless, investigation of the cellular mechanisms directed at removal of ribonucleotides from DNA has only recently attracted considerable attention. The first line of defense comes from the innate structural features of DNA polymerases themselves. Active sites of most DNA polymerases contain a so-called “steric gate” residue that plays a major role in rNMP exclusion by colliding with the 2′-hydroxyl of an incoming ribonucleotide [58]. The steric gate residue of pol V is Y11 in the UmuC subunit of the polymerase and is among the least efficient barriers against ribonucleotide incorporation since wild-type pol V readily incorporates ribonucleotides into DNA [14]. Substitution of Y11 with a much smaller alanine residue takes sugar indiscretion of the variant polymerase to extremes, since when presented with both types of deoxy- and ribonucleotides, UmuC_Y11A often selects rNTPs during primer extension [14].

Biochemical characterization of UmuC_Y11A and wild-type pol V revealed that besides the differences in sugar selectivity, other properties of the polymerases are similar [14], [23]. Despite a virtually identical base-substitution fidelity *in vitro*, the mutability of the strains expressing *umuC*_Y11A is quite low compared to wild-type pol V [23]. The difference in spontaneous mutagenesis is explained by the extent of the accumulation of errant rNMPs into genomic DNA, and the subsequent actions of repair pathways directed at rNMP removal [11]. Although triggered by the presence of nucleotides with the wrong sugar, activation of these pathways also results in the removal of the deoxyribonucleotides base mispairs which happen to lie inside the rNMP repair “patch” [11]. The connection between rNMP repair and levels of spontaneous mutagenesis therefore provides a unique opportunity to elucidate various repair mechanisms aimed at sanitization of errantly incorporated NTPs from the *E.coli* genome. Thus, changes in the extent of *umuC*_Y11A-dependent spontaneous mutagenesis compared to wild-type pol V mutagenesis should identify pathways for rNMP removal. Indeed, using such an approach, we have recently demonstrated that the main pathway directed at rNMP excision involves the nicking action of RNase HII [11] and subsequent strand-displacement DNA synthesis by pol I (unpublished observations). In the present study, we have analyzed the contribution of mismatch repair (MMR), base-excision repair (BER) and nucleotide excision repair (NER) to ribonucleotide repair in *E.coli*.

Although *umuC*_Y11A is expected to frequently incorporate ribo-UMP into DNA, we found no evidence that deletion of *ung*, *xth* or *nfo*, all of which lead to defects in the uracil glycosylase-mediated BER pathway, has any effect on ribonucleotide repair in *E.coli* (Fig. 1). Such observations are, therefore, consistent with the limited ability of the uracil glycosylase to remove rU compared to dU *in vitro* [59]–[61]. Furthermore, given the fact that the level of *umuC*_Y11A-dependent mutagenesis observed when *rnhA*, *rnhB* and NER are all inactivated was even higher than with wild-type pol V (Fig. 3), it seems unlikely that another, as yet unidentified, BER enzyme(s) might contribute to RER, but it cannot be formally excluded.

When compared to the level of mutagenesis exhibited by wild type pol V, mismatch repair-deficient Δ*mutL*, Δ*mutH* and Δ*mutS* cells all exhibited higher levels of *umuC*_Y11A-dependent mutagenesis (Fig. 1). This was initially assumed to reflect the participation of MMR in the removal of ribonucleotides incorporated by *umuC*_Y11A, especially if the base was also mispaired [13]. However, there was no further increase in *umuC*_Y11A mutagenesis in the MMR^−^ strains upon deletion of RNase HII (Fig. 2). Based on our recent finding revealing that the extent of mutagenesis in *rnhB* ^+^ MMR-deficient strains is dependent upon pol I (unpublished observations), we hypothesize that the increase in mutagenesis observed in the *rnhB* ^+^ *umuC*_Y11A MMR^−^ strains actually reflects persisting transition errors which are made by pol I during RNase HII-initiated ribonucleotide repair and which otherwise would be subjected to repair in MMR^+^ cells (Fig. 2). Overall, our data suggest that even though MMR is able to remove mispaired ribonucleotides from DNA, it has a limited (if any), role in prokaryotic RER *in vivo*. Conversely, it has recently been shown that RNase HII-dependent RER plays a significant role in MMR in eukaryotes by providing the strand-discrimination signal that identifies the newly synthesized DNA [62], [63].

In contrast to MMR and BER, our present study strongly implicates NER as a backup mechanism for ribonucleotide repair in prokaryotes (Fig. 1). The *in vivo* data suggest that similar to the RNase HI-dependent pathway, NER is not a primary mechanism of rNMP repair in cells with a functional RNase HII-initiated RER pathway, but plays an important role in the absence of RNase HII. Furthermore, it appears that in Δ*rnhB* cells both the NER proteins and RNase HI are required for efficient ribonucleotide repair (Fig. 3). While RNase HI specializes in removal of longer RNA fragments, the UvrABC endonuclease is able to eliminate isolated rNMPs, as well as to compete with RNase HI for removal of several sequential ribonucleotides.

In general, it is assumed that NER is the major defense mechanism against bulky DNA adducts, although it is also known to repair relatively minor DNA modifications, such as apurinic sites (reviewed in [40], [41]) and we report here that even misincorporated ribonucleotides that only differ by a single 2′-OH from their deoxyribose counterparts, are also substrates for NER. UvrB/C dependent incisions are made 8 bp 5′ and 4–5 bp 3′ to the ribonucleotide generating a ribonucleotide containing fragment of ∼12–13 bases (Fig. 4 & 6), which is identical to that obtained with DNA damage-mediated NER in *E.coli* [40].

So how is the ribose moiety recognized as a “lesion”? Based on the analysis of the structure and conformation of the diverse set of DNA lesions that are repaired by the NER machinery, it has been suggested that UvrA_2_B complex is not targeted by nucleotide damage *per se*, but rather by damaged-induced conformational changes in DNA; the more the DNA helix deviates from the canonical B-form conformation, the more efficient the NER [40], [41]. This is supported by the fact that NER is much more efficient within the context of distorting base mispairs (e.g., Fig. 3A). Similarly, we show that the ribonucleotide excision activity of UvrABC endonuclease varies depending on the number of ribonucleotides incorporated into DNA, the presence of base mismatches, and the type and number of mispaired bases, suggesting that it the distortion of the DNA around the ribonucleotide that helps target it for NER (Fig. 4). However, we also observed significant incision of a single correctly-paired rNMP embedded in DNA, which is unlikely to cause a major conformational change in the local sequence surrounding the ribonucleotide [3]. Since the ribonucleotide moiety provides a negative electrostatic potential and offers new hydrogen bonding opportunities compared to the deoxynucleotide, it is possible that the local differences between an embedded ribo- vs. deoxynucleotide might lead to NER recognition. Clearly, the mechanisms underlying ribonucleotide recognition by the NER complex is a topic that should be investigated further.

**Figure 7:**
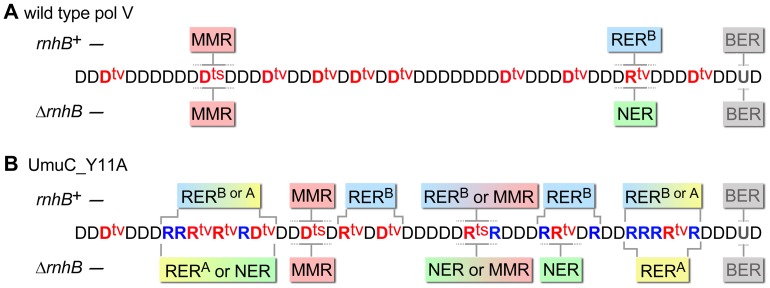
Various DNA repair pathways compete, cooperate, or substitute for each other in order to sanitize the *E.coli* chromosome from mispairs, uracils and incorporated rNMPs. The cartoon helps to explain why spontaneous mutagenesis induced by wild-type pol V (A) differs from Y11A_UmuC-dependent mutagenesis (B) and illustrates the respective roles of MMR (red box), RER^A^ (stands for RNase HI-initiated ribonucleotide excision repair and is indicated in yellow), RER^B^ (RNase HII-initiated ribonucleotide excision repair, indicated in blue), NER (green), and BER (grey) (The competing pathways are indicated by boxes with gradient colors). Misincorporated nucleotides are shown in red (where ts are transitions, tv are transversions), correctly paired ribonucleotides are indicated in blue. For simplicity, all the transversions are shown refractory to MMR and NER while in reality they could be repaired by both pathways although less efficiently than transitions. Both wild-type and mutant pol V make frequent base-substitution errors. The transition mutations are rapidly repaired by MMR, while any errant ribonucleotides (correctly-paired or mispaired) are also efficiently removed by RER^B^. Ung-dependent BER only operates on dU, not rU, incorporated into the DNA and therefore has no role in RER. In contrast, NER is able to remove rNMPs misincorporated by either wild-type or mutant pol V. Since *umuC*_Y11A is able to incorporate multiple consecutive rNMPs into DNA, RER involving RNase HI is limited to strains expressing the pol V variant. RER^B^ is normally required for highly efficient removal of errant ribonucleotides, however, in its absence the role of NER and RNase HI becomes apparent. The fact that the level of spontaneous mutagenesis in strains expressing *umuC*_Y11A with an “unlocked” sugar steric gate is 90% lower than mutagenesis in strains harboring wild-type pol V, implies that numerous errant ribonucleotides are very efficiently excised by the collaborative actions of rNMP-specific repair pathways which concomitantly remove mispaired dNMPs positioned within the repair patch (such as for example two D^tv^s in the panel B). In the absence of RNase HI, RNase HII and NER proteins, the majority of the misincorporated rNMPs remains embedded in the chromosomal DNA. As a result, spontaneous mutagenesis in the Δ*rnhA* Δ*rnhB* Δ*uvrA* strain expressing *umuC*_Y11A is higher than in the isogenic strain expressing wild-type pol V.

In summary, we show here that a complex network of DNA repair mechanisms is involved in cleansing chromosomal DNA of misincorporated ribonucleotides (Fig. 7). RER initiated by RNase HII plays the leading role in removing isolated rNMPs. When this pathway is overloaded, or inactivated, prominent backup roles are assumed by RNase HI and NER proteins. In general, RNase HI facilitates the removal of stretches of ribonucleotides 4 bp or more in length, while NER can excise single and poly-ribonucleotides embedded in DNA. As a consequence, both RNase HI and NER proteins help to reduce genomic instability generated though errant ribonucleotide misincorporation.

## Materials and Methods

### Bacterial strains

Most of the E. coli K-12 strains used in this study are derivatives of RW698 (full genotype: recA730 lexA51(Def) ΔdinB61::ble ΔumuDC596::ermGT thr-1 araD139 Δ(gpt-proA)62 lacY1 tsx-33 glnV44 galK2 hisG4 rpsL31 xyl-5 mtl-1 argE3 thi-1 sulA211) [11]. Repair-deficient “KEIO” strains were obtained from the E.coli Genetic Stock Center and isogenic derivatives of RW698 were generated via generalized transduction using P1vir [64] (Table 1). Where noted, Kan^S^ strains were obtained by transforming cells with the temperature sensitive ampicillin and chloramphenicol resistant plasmid, pCP20, which expresses the FLP recombinase [65]. Transformants were selected on LB plates containing the appropriate antibiotics at 25°C and subsequently re-streaked on LB plates lacking ampicillin, chloramphenicol and kanamycin and incubated overnight at 43°C. Colonies from these plates were subsequently confirmed to be ampicillin, chloramphenicol and kanamycin sensitive at 37°C.

**Table 1:** *E.coli* strains used in this study.

Strain	Relevant Genotype	Source or Reference
ES1484	*mutL218*::Tn*10*	*E.coli* Genetic Stock Center
JW4128	Δ*mutL720*::Kan	*E.coli* Genetic Stock Center
JW2799	Δ*mutH756*::Kan	*E.coli* Genetic Stock Center
JW2703	Δ*mutS738*::Kan	*E.coli* Genetic Stock Center
JW2146	Δ*nfo786*::Kan	*E.coli* Genetic Stock Center
JW2564	Δ*ung748*::Kan	*E.coli* Genetic Stock Center
JW1738	Δ*xthA747*::Kan	*E.coli* Genetic Stock Center
JW4019	Δ*uvrA753*::Kan	*E.coli* Genetic Stock Center
RW1058	*uvrB5 nadA57*::Tn*10*	LGI Stocks
CS5540	Δ*cho*::*cat*	[35]
JW3786	Δ*uvrD769*::Kan	*E.coli* Genetic Stock Center
RW698^a^	*recA730 lexA51*(Def) Δ*(umuDC)596*::*ermGT* Δ*dinB61*::*ble*	[11]
RW1044^a^	*recA730 lexA51*(Def) Δ*(umuDC)596*::*ermGT* Δ*dinB61*::*ble* Δ*rnhA339*::*cat*	[11]
RW838^a^	*recA730 lexA51*(Def) Δ*(umuDC)596*::*ermGT* Δ*dinB61*::*ble* Δ*rnhB782*::Kan	[11]
RW1092^a^	*recA730 lexA51*(Def) Δ*(umuDC)596*::*ermGT* Δ*dinB61*::*ble* Δ*rnhA339*::*cat* Δ*rnhB782*::Kan	[11]
RW710^a^	*recA730 lexA51*(Def) Δ*(umuDC)596*::*ermGT* Δ*dinB61*::*ble mutL211*::Tn*5*	[25]
RW1236^a^	*recA730 lexA51*(Def) Δ*(umuDC)596*::*ermGT* Δ*dinB61*::*ble* Δ*mutL720*::Kan	RW698 x P1. JW4128
RW1104^a^	*recA730 lexA51*(Def) Δ*(umuDC)596*::*ermGT* Δ*dinB61*::*ble* Δ*mutH756*::Kan	RW698 x P1. JW2799
RW1192^a^	*recA730 lexA51*(Def) Δ*(umuDC)596*::*ermGT* Δ*dinB61*::*ble* Δ*mutS738*::Kan	RW698 x P1. JW2703
RW942^a^	*recA730 lexA51*(Def) Δ*(umuDC)596*::*ermGT* Δ*dinB61*::*ble* Δ*rnhB782*::Kan *mutL218*::Tn*10*	RW838 x P1. ES1484
RW970^a^	*recA730 lexA51*(Def) Δ*(umuDC)596*::*ermGT* Δ*dinB61*::*ble* Δ*rnhB782*	Kan^S^ derivative of RW838
RW1056^a^	*recA730 lexA51*(Def) Δ*(umuDC)596*::*ermGT* Δ*dinB61*::*ble* Δ*rnhB782* Δ*mutL720*::Kan	RW970 x P1. JW4128
RW1062^a^	*recA730 lexA51*(Def) Δ*(umuDC)596*::*ermGT* Δ*dinB61*::*ble* Δ*rnhB782* Δ*mutH756*::Kan	RW970 x P1. JW2799
RW1194^a^	*recA730 lexA51*(Def) Δ*(umuDC)596*::*ermGT* Δ*dinB61*::*ble* Δ*rnhB782* Δ*mutS738*::Kan	RW970 x P1. JW2703
RW1238^a^	*recA730 lexA51*(Def) Δ*(umuDC)596*::*ermGT* Δ*dinB61*::*ble* Δ*nfo786*::Kan	RW698 x P1. JW2146
RW1248^a^	*recA730 lexA51*(Def) Δ*(umuDC)596*::*ermGT* Δ*dinB61*::*ble* Δ*ung748*::Kan	RW698 x P1. JW2564
RW1240^a^	*recA730 lexA51*(Def) Δ*(umuDC)596*::*ermGT* Δ*dinB61*::*ble* Δ*xthA747*::Kan	RW698 x P1. JW1738
RW1076^a^	*recA730 lexA51*(Def) Δ*(umuDC)596*::*ermGT* Δ*dinB61*::*ble* Δ*rnhB782* Δ*nfo786*::Kan	RW970 x P1. JW2146
RW1074^a^	*recA730 lexA51*(Def) Δ*(umuDC)596*::*ermGT* Δ*dinB61*::*ble* Δ*rnhB782* Δ*ung748*::Kan	RW970 x P1. JW2564
RW1078^a^	*recA730 lexA51*(Def) Δ*(umuDC)596*::*ermGT* Δ*dinB61*::*ble* Δ*rnhB782* Δ*xthA747*::Kan	RW970 x P1. JW1738
RW902^a^	*recA730 lexA51*(Def) Δ*(umuDC)596*::*ermGT* Δ*dinB61*::*ble* Δ*uvrA753*::Kan	[11]
RW1094^a^	*recA730 lexA51*(Def) Δ*(umuDC)596*::*ermGT* Δ*dinB61*::*ble uvrB5 nadA57*::Tn*10*	RW698 x P1. RW1058
RW906^a^	*recA730 lexA51*(Def) Δ*(umuDC)596*::*ermGT* Δ*dinB61*::*ble* Δ*uvrC759*::Kan	[11]
RW908^a^	*recA730 lexA51*(Def) Δ*(umuDC)596*::*ermGT* Δ*dinB61*::*ble* Δ*cho*::cat	RW698 x P1. CS5540
RW1184^a^	*recA730 lexA51*(Def) Δ*(umuDC)596*::*ermGT* Δ*dinB61*::*ble* Δ*uvrD769*::Kan	RW698 x P1. JW3786
RW990^a^	*recA730 lexA51*(Def) Δ*(umuDC)596*::*ermGT* Δ*dinB61*::*ble* Δ*rnhB782* Δ*uvrA753*::Kan	RW970 x P1. JW4019
RW1096^a^	*recA730 lexA51*(Def) Δ*(umuDC)596*::*ermGT* Δ*dinB61*::*ble* Δ*rnhB782 uvrB5 nadA57*::Tn*10*	RW970 x P1. RW1058
RW1068^a^	*recA730 lexA51*(Def) Δ*(umuDC)596*::*ermGT* Δ*dinB61*::*ble* Δ*rnhB782* Δ*uvrC759*::Kan	RW970 x P1. JW1898
RW1070^a^	*recA730 lexA51*(Def) Δ*(umuDC)596*::*ermGT* Δ*dinB61*::*ble* Δ*rnhB782* Δ*cho*::cat	RW970 x P1. CS5540
RW1186^a^	*recA730 lexA51*(Def) Δ*(umuDC)596*::*ermGT* Δ*dinB61*::*ble* Δ*rnhB782* Δ*uvrD769*::Kan	RW970 x P1. JW3786
RW1182^a^	*recA730 lexA51*(Def) Δ*(umuDC)596*::*ermGT* Δ*dinB61*::*ble* Δ*rnhA339*::*cat* Δ*rnhB782*	Kan^S^ derivative of RW1092
RW1190^a^	*recA730 lexA51*(Def) Δ*(umuDC)596*::*ermGT* Δ*dinB61*::*ble* Δ*rnhA339*::*cat* Δ*rnhB782* Δ*uvrA753*::Kan	RW1182 x P1. JW4019

a : *thr-1 araD139* Δ*(gpt-proA)62 lacY1 tsx-33 glnV44 galK2 hisG4 rpsL31 xyl-5 mtl-1 argE3 thi-1 sulA211.*

The following antibiotics were used for selection; Zeocin (25 µg/ml), Kanamycin (50 µg/ml), Tetracycline (15 µg/ml), Chloramphenicol (20 µg/ml), and Ampicillin (100 µg/ml), Spectinomycin (50 µg/ml).

### Plasmids

The low-copy-number spectinomycin resistant plasmids pRW134 and pJM963 which encode *E.coli* wild-type UmuC and the *umuC*_Y11A variant, respectively, along with UmuD′ [23] are derived from pGB2 [66] and express the UmuD′C proteins at close to physiological levels from their native promoter [23]. Bacteria harboring plasmids were grown in LB media containing appropriate 50 µg/ml spectinomycin.

### Quantitative spontaneous mutagenesis assay

Cells transformed with the vector plasmid, pGB2, or the low-copy number plasmid pRW134 expressing wild-type pol V, or pJM963 expressing the *umuC*_Y11A variant [23] were grown overnight at 37°C in LB media plus appropriate antibiotics. The next day, cultures were centrifuged and resuspended in an equal volume of SM buffer [64]. To determine the number of spontaneously arising histidine mutants on the plate, the cell cultures were seeded on the Davis and Mingioli minimal agar plates [67] plus glucose (0.4% wt/vol); agar (1.0% wt/vol); proline, threonine, valine, leucine, and isoleucine (all at 100 µg/ml); thiamine (0.25 µg/ml); and either no histidine, or histidine (1 µg/ml). On the plates containing no histidine, only pre-existing His^+^ mutants grew to form colonies. When ∼4×10^7^ bacteria were seeded on the 1 µg/ml histidine, they grew to form a lawn, concomitantly exhausting the low level of histidine. Spontaneously arising His^+^ mutants grew up through the lawn and were counted after 4 days incubation at 37°C. Spontaneous mutagenesis is expressed as a frequency (mutants per plate), because the number of mutants arising on the plate is independent of the number of cells plated, but is, instead, dependent upon the limiting amount of nutrient (histidine) in the plate [68]. The *relative* extent of *umuC*_Y11A mutagenesis was calculated by first subtracting the number of pre-existing His^+^ mutants (mutants arising on the plates lacking histidine) and subsequently dividing the number of spontaneously arising mutants on the *umuC*_Y11A (pJM963) plates by the number of spontaneously arising mutants on the wild-type pol V (pRW134) plates. The data reported in Figs. 1 and 3 represent the average number of His^+^ mutants from at least 3 separate experiments (± standard error of the mean [SEM]).

### Spectra of spontaneous base-pair substitutions in the *E.coli rpoB* gene

The mutation spectra were generated using the *rpoB*/Rif^R^ mutagenesis assay [69], [70]. A single pair of oligonucleotide primers can be used for PCR amplification and a single primer for DNA sequencing because 88% of all *rpoB* mutations are localized in the central 202 bp region of the gene [69]. *E. coli* strains RW710 [relevant genotype: *lexA*(Def) *recA730* Δ*dinB* Δ*umuDC* Δ*mutL*], RW942 [relevant genotype: *lexA*(Def) *recA730* Δ*dinB* Δ*umuDC* Δ*mutL* Δ*rnhB* ], RW698 [relevant genotype: *lexA*(Def) *recA730* Δ*dinB* Δ*umuDC*], and RW838 [relevant genotype: *lexA*(Def) *recA730* Δ*dinB* Δ*umuDC* Δ*rnhB* ], harboring the *umuC*_Y11A plasmid, pJM963 were diluted from a frozen stock cultures such that the initial inoculum contained <1000 viable cells. Cultures were grown in LB for 24 h at 37°C and appropriate dilutions spread on an LB agar plate containing 100 µg/ml rifampicin. Individual independent Rif^R^ colonies were picked from the plate using a pipette tip and subjected to PCR in a 96-well micro-titer plate. An <1 kb central region of the *rpoB* gene was amplified using the PCR primers RpoB1: 5′-CAC A**C**G GCA TCT GGT TGA TAC AG-3′ and RpoF1: 5′-TGG CGA AAT GGC GGA AAA C-3 by denaturation at 95°C for 3 min, followed by 30 cycles of 94°C for 30 s, 1 min at 59°C, 2 min at 72°C, followed by a final extension step at 72°C for 7 min. The nucleotide sequence of the ∼200 bp target region of *rpoB* in each PCR amplicon was determined by Beckman Coulter Genomics (Danvers, MA) using WOG923AP01 primer (5′-CAG TTC CGC GTT GGC CTG-3′). Only base-pair substitutions occurring between positions 1516 and 1717 of the *rpoB* gene were considered during data analysis. Nucleotide sequences obtained were aligned and analyzed using the ClustalW multiple sequence alignment program (Hinxton, UK). Rates for forward mutations to rifampicin resistance (mutations in *rpoB*) were determined as previously described [25].

### Excision of various DNA substrates by the UvrABC complex

The *Bacillus caldotenax* UvrA and UvrB proteins and the *Thermatoga maritima* UvrC protein were purified as previously described [39], [71]. *E. coli* RNase HII was purchased from New England Biolabs (Ipswich, MA, USA).

All oligonucleotides were synthesized by Lofstrand Laboratories (Gaithersburg, MD) and gel purified prior to use. The basic sequence of the 50-mer template is: 5′-GAC TAC GTA CTG TTA CGG CTC CAT c**aa** taC CGC AAT CAG GCC AGA TCT GC-3′. The lowercase letters indicate sites at which substrate dNMPs were replaced by rNMPs. Substrates with a single rAMP (first underlined a on the 5′ side), two consecutive rAMPs (shown in bold), or five rNMPs were tested. A substrate with the site-specifically placed fluorescein adduct (fT) [39] was used as a control for the activity of NER proteins and has the same sequence except that the aa bases (shown in bold) were replaced with the [fT]C sites. The fT-containing oligonucleotide and the DNA-RNA-DNA hybrids were 5′- or 3′-^32^P end-labeled and annealed with either completely complementary DNA strands, or with the DNA strands containing one or two mispaired bases. In the case of the fluorescein-adducted template, the damaged T was either correctly paired with A, or mispaired with C. In the case of the DNA-RNA-DNA hybrids, either the 5′A, or both As shown in bold were correctly paired with Ts or mispaired with one, or two, Cs or As. Hybridization was performed at a 1.5 molar excess of the unlabeled strand by heating in an annealing buffer (50 mM Tris-HCl (pH 8), 5 mM MgCl_2_, 50 µg/ml BSA, 1.42 mM 2-mercaptoethanol) for 10 min at 100°C followed by slow cooling to room temperature. Prior to initiation of the incision assay, the UvrABC proteins were diluted from stock solutions and preheated for 10 min at 55°C. The 10 nM DNA substrates were incubated with UvrA (40 nM), UvrB (200 nM), and UvrC (100 nM) proteins for 1 hour at 55°C in the presence of 1 mM ATP in a 1× reaction buffer (10 mM Tris, pH 7.5, 10 mM KCl, 2 mM MgCl_2_, 1 mM DTT, 0.2 mM ATP). Cleavage of the 10 nM DNA substrates by RNase HII was performed according to the manufacturer's instructions. Reactions were terminated by the addition of 2× loading buffer (97% formamide, 10 mM EDTA, 0.1% xylene cyanol, 0.1% bromophenol blue) and the incision products were analyzed on a 15% denaturing polyacrylamide gel. The extent of incision was determined for each substrate and expressed as a percentage of radioactivity in the cleaved products relative to the total signal. Data shown below the gels are the mean values calculated from at least two independent experiments.

## Supporting Information

Figure S1 *In vitro* cleavage reactions catalyzed by NER or RNase HII using various DNA-RNA-DNA hybrid templates. The 50-mer duplexes (10 nM) in which the DNA-only strand that is complementary to the rNMP-containing oligonucleotide was 5′ end-labeled (indicated by *), were incubated with either RNase HII, or with the NER proteins and the reaction products were analyzed as described in the legends to Figs. 4 & 5. The sequence of the 50-mer template containing a single rNTP (indicated as “y”) is: 5′-GAC TAC GTA CTG TTA CGG CTC CAT CyA TAC CGC AAT CAG GCC AGA TCT GC-3′. The local sequence context surrounding the ribonucleotide is shown below the gel. The figure demonstrates that the NER and RNase HII mediated incisions observed in Figs. 4 & 5 are specific to the ribonucleotide containing strand, as no incisions are observed on the complementary DNA strand, irrespective of whether it is correctly or incorrectly paired.
(PDF)

Table S1 The type of base-pair substitutions generated in the *E.coli rpoB* gene were determined for *recA730 lexA*(Def) Δ*dinB* Δ*umuDC* strains expressing *umuC*_Y11A which are proficient- or deficient- in MMR and RNase HII- mediated RER. The data for the *rnhB* ^+^ *mutL* strain (RW710) are taken from [13] and are shown for direct comparison with the data for the isogenic Δ*rnhB mutL* strain (RW942). 290–375 Rif^R^ mutants were analyzed in the various strain backgrounds. The spectra of mutations obtained in the *rnhB* ^+^ strain represents mutagenic events generated by the DNA polymerase that participates in RER, while the spectra of mutations obtained in the Δ*rnhB* strains represents persisting mutagenic events in the absence of RER and which are promoted by *umuC*_Y11A. In both the *rnhB* ^+^ and Δ*rnhB* strains deficient in MMR, the spectra are dominated by transitions, but the mutagenic hotspots differ depending on the presence, or absence, of RNase HII. Furthermore the Δ*rnhB mutL* strain exhibits an ∼4-fold increase in the number of transversion mutations that are characteristic of pol V. In the *mutL* ^+^ strains, transitions are efficiently repaired by MMR and as a consequence, the spectra are dominated by transversion events. Similar to the MMR-deficient strains, the location of the mutagenic hotspots depends upon the presence, or absence of RNase HII and are indicative of mutagenic events generated by two different DNA polymerases working in the presence or absence of RER.
(PDF)

## References

[1] Lindahl T (1993) Instability and decay of the primary structure of DNA. Nature 362: 709–715. 8469282 10.1038/362709a0

[2] Wahl MC, Sundaralingam M (2000) B-form to A-form conversion by a 3′-terminal ribose: crystal structure of the chimera d(CCACTAGTG)r(G). Nucleic Acids Res 28: 4356–4363. 11058136 10.1093/nar/28.21.4356 PMC113134

[3] DeRose EF, Perera L, Murray MS, Kunkel TA, London RE (2012) Solution structure of the Dickerson DNA dodecamer containing a single ribonucleotide. Biochemistry 51: 2407–2416. 22390730 10.1021/bi201710q PMC3743102

[4] Nick McElhinny SA, Kumar D, Clark AB, Watt DL, Watts BE, et al (2010) Genome instability due to ribonucleotide incorporation into DNA. Nat Chem Biol 6: 774–781. 20729855 10.1038/nchembio.424 PMC2942972

[5] Reijns MA, Rabe B, Rigby RE, Mill P, Astell KR, et al (2012) Enzymatic removal of ribonucleotides from DNA is essential for mammalian genome integrity and development. Cell 149: 1008–1022. 22579044 10.1016/j.cell.2012.04.011 PMC3383994

[6] Lazzaro F, Novarina D, Amara F, Watt DL, Stone JE, et al (2012) RNase H and postreplication repair protect cells from ribonucleotides incorporated in DNA. Mol Cell 45: 99–110. 22244334 10.1016/j.molcel.2011.12.019 PMC3262129

[7] Hiller B, Achleitner M, Glage S, Naumann R, Behrendt R, et al (2012) Mammalian RNase H2 removes ribonucleotides from DNA to maintain genome integrity. J Exp Med 209: 1419–1426. 22802351 10.1084/jem.20120876 PMC3409502

[8] Zheng L, Shen B (2011) Okazaki fragment maturation: nucleases take centre stage. J Mol Cell Biol 3: 23–30. 21278448 10.1093/jmcb/mjq048 PMC3030970

[9] Rydberg B, Game J (2002) Excision of misincorporated ribonucleotides in DNA by RNase H (type 2) and FEN-1 in cell-free extracts. Proc Natl Acad Sci U S A 99: 16654–16659. 12475934 10.1073/pnas.262591699 PMC139199

[10] Sparks JL, Chon H, Cerritelli SM, Kunkel TA, Johansson E, et al (2012) RNase H2-Initiated Ribonucleotide Excision Repair. Mol Cell 47: 980–986. 22864116 10.1016/j.molcel.2012.06.035 PMC3470915

[11] McDonald JP, Vaisman A, Kuban W, Goodman MF, Woodgate R (2012) Mechanisms employed by *Escherichia coli* to prevent ribonucleotide incorporation into genomic DNA by pol V. PLoS Genet 8: e1003030. 23144626 10.1371/journal.pgen.1003030 PMC3493448

[12] Kim N, Huang SN, Williams JS, Li YC, Clark AB, et al (2011) Mutagenic processing of ribonucleotides in DNA by yeast topoisomerase I. Science 332: 1561–1564. 21700875 10.1126/science.1205016 PMC3380281

[13] Shen Y, Koh KD, Weiss B, Storici F (2012) Mispaired rNMPs in DNA are mutagenic and are targets of mismatch repair and RNases H. Nat Struc Mol Biol 19: 98–104. 10.1038/nsmb.2176 22139012

[14] Vaisman A, Kuban W, McDonald JP, Karata K, Yang W, et al (2012) Critical amino acids in *Escherichia coli* responsible for sugar discrimination and base-substitution fidelity. Nucleic Acids Res 40: 6144–6157. 22422840 10.1093/nar/gks233 PMC3401427

[15] Tang M, Shen X, Frank EG, O'Donnell M, Woodgate R, et al (1999) UmuD′_2_C is an error-prone DNA polymerase, *Escherichia coli*, DNA pol V. Proc Natl Acad Sci U S A 96: 8919–8924. 10430871 10.1073/pnas.96.16.8919 PMC17708

[16] Jiang Q, Karata K, Woodgate R, Cox MM, Goodman MF (2009) The active form of DNA polymerase V is UmuD′_2_C-RecA-ATP. Nature 460: 359–363. 19606142 10.1038/nature08178 PMC2731490

[17] Shinagawa H, Iwasaki H, Kato T, Nakata A (1988) RecA protein-dependent cleavage of UmuD protein and SOS mutagenesis. Proc Natl Acad Sci U S A 85: 1806–1810. 3126496 10.1073/pnas.85.6.1806 PMC279868

[18] Woodgate R, Ennis DG (1991) Levels of chromosomally encoded Umu proteins and requirements for in vivo UmuD cleavage. Mol Gen Genet 229: 10–16. 1654503 10.1007/BF00264207

[19] Frank EG, Gonzalez M, Ennis DG, Levine AS, Woodgate R (1996) In vivo stability of the Umu mutagenesis proteins: a major role for RecA. J Bacteriol 178: 3550–3556. 8655553 10.1128/jb.178.12.3550-3556.1996 PMC178125

[20] Frank EG, Ennis DG, Gonzalez M, Levine AS, Woodgate R (1996) Regulation of SOS mutagenesis by proteolysis. Proc Natl Acad Sci U S A 93: 10291–10296. 8816793 10.1073/pnas.93.19.10291 PMC38377

[21] Sweasy JB, Witkin EM, Sinha N, Roegner-Maniscalco V (1990) RecA protein of *Escherichia coli* has a third essential role in SOS mutator activity. J Bacteriol 172: 3030–3036. 2188949 10.1128/jb.172.6.3030-3036.1990 PMC209104

[22] Fijalkowska IJ, Dunn RL, Schaaper RM (1997) Genetic requirements and mutational specificity of the *Escherichia coli* SOS mutator activity. J Bacteriol 179: 7435–7445. 9393709 10.1128/jb.179.23.7435-7445.1997 PMC179695

[23] Kuban W, Vaisman A, McDonald JP, Karata K, Yang W, et al (2012) *Escherichia coli* UmuC active site mutants: effects on translesion DNA synthesis, mutagenesis and cell survival. DNA Repair 11: 726–732. 22784977 10.1016/j.dnarep.2012.06.005 PMC3419331

[24] Watanabe-Akanuma M, Woodgate R, Ohta T (1997) Enhanced generation of A:T->T:A transversions in a *recA730 lexA51*(Def) mutant of *Escherichia coli* . Mutat Res 373: 61–66. 9015154 10.1016/s0027-5107(96)00189-3

[25] Curti E, McDonald JP, Mead S, Woodgate R (2009) DNA polymerase switching: effects on spontaneous mutagenesis in *Escherichia coli* . Mol Microbiol 71: 315–331. 19019142 10.1111/j.1365-2958.2008.06526.x PMC2680738

[26] Kouzminova EA, Kuzminov A (2008) Patterns of chromosomal fragmentation due to uracil-DNA incorporation reveal a novel mechanism of replication-dependent double-stranded breaks. Mol Microbiol 68: 202–215. 18312272 10.1111/j.1365-2958.2008.06149.x

[27] Kow YW, Wallace SS (1985) Exonuclease III recognizes urea residues in oxidized DNA. Proc Natl Acad Sci U S A 82: 8354–8358. 3001698 10.1073/pnas.82.24.8354 PMC390914

[28] Cunningham RP, Saporito SM, Spitzer SG, Weiss B (1986) Endonuclease IV (*nfo*) mutant of *Escherichia coli* . J Bacteriol 168: 1120–1127. 2430946 10.1128/jb.168.3.1120-1127.1986 PMC213611

[29] Sandigursky M, Freyer GA, Franklin WA (1998) The post-incision steps of the DNA base excision repair pathway in *Escherichia coli*: studies with a closed circular DNA substrate containing a single U:G base pair. Nucleic Acids Res 26: 1282–1287. 9469838 10.1093/nar/26.5.1282 PMC147386

[30] Sanderson RJ, Bennett SE, Sung JS, Mosbaugh DW (2001) Uracil-initiated base excision DNA repair synthesis fidelity in human colon adenocarcinoma LoVo and *Escherichia coli cell* extracts. Prog Nuc Acid Res Mol Biol 68: 165–188. 10.1016/s0079-6603(01)68098-x 11554295

[31] Hou EW, Prasad R, Asagoshi K, Masaoka A, Wilson SH (2007) Comparative assessment of plasmid and oligonucleotide DNA substrates in measurement of *in vitro* base excision repair activity. Nucleic Acids Res 35: e112. 17720705 10.1093/nar/gkm639 PMC2034467

[32] Fernández de Henestrosa AR, Ogi T, Aoyagi S, Chafin D, Hayes JJ, et al (2000) Identification of additional genes belonging to the LexA-regulon in *Escherichia coli* . Mol Microbiol 35: 1560–1572. 10760155 10.1046/j.1365-2958.2000.01826.x

[33] Lu AL, Clark S, Modrich P (1983) Methyl-directed repair of DNA base-pair mismatches *in vitro* . Proc Natl Acad Sci U S A 80: 4639–4643. 6308634 10.1073/pnas.80.15.4639 PMC384099

[34] Yamaguchi M, Dao V, Modrich P (1998) MutS and MutL activate DNA helicase II in a mismatch-dependent manner. J Biol Chem 273: 9197–9201. 9535910 10.1074/jbc.273.15.9197

[35] Moolenaar GF, van Rossum-Fikkert S, van Kesteren M, Goosen N (2002) Cho, a second endonuclease involved in *Escherichia coli* nucleotide excision repair. Proc Natl Acad Sci U S A 99: 1467–1472. 11818552 10.1073/pnas.032584099 PMC122214

[36] Crowley DJ, Hanawalt PC (2001) The SOS-dependent upregulation of *uvrD* is not required for efficient nucleotide excision repair of ultraviolet light induced DNA photoproducts in *Escherichia coli* . Mutat Res 485: 319–329. 11585364 10.1016/s0921-8777(01)00068-4

[37] Jiang G, Skorvaga M, Van Houten B, States JC (2003) Reduced sulfhydryls maintain specific incision of BPDE-DNA adducts by recombinant thermoresistant *Bacillus caldotenax* UvrABC endonuclease. Prot Exp Pur 31: 88–98. 10.1016/s1046-5928(03)00137-2 12963345

[38] Skorvaga M, Theis K, Mandavilli BS, Kisker C, Van Houten B (2002) The β-hairpin motif of UvrB is essential for DNA binding, damage processing, and UvrC-mediated incisions. J Biol Chem 277: 1553–1559. 11687584 10.1074/jbc.M108847200

[39] Croteau DL, DellaVecchia MJ, Wang H, Bienstock RJ, Melton MA, et al (2006) The C-terminal zinc finger of UvrA does not bind DNA directly but regulates damage-specific DNA binding. J Biol Chem 281: 26370–26381. 16829526 10.1074/jbc.M603093200 PMC2396232

[40] Van Houten B (1990) Nucleotide excision repair in *Escherichia coli* . Microbiol Rev 54: 18–51. 2181258 10.1128/mr.54.1.18-51.1990 PMC372757

[41] Truglio JJ, Croteau DL, Van Houten B, Kisker C (2006) Prokaryotic nucleotide excision repair: the UvrABC system. Chem Rev 106: 233–252. 16464004 10.1021/cr040471u

[42] Lin JJ, Sancar A (1990) Reconstitution of nucleotide excision nuclease with UvrA and UvrB proteins from *Escherichia coli* and UvrC protein from *Bacillus subtilis* . J Biol Chem 265: 21337–21341. 2174442

[43] Ruan Q, Liu T, Kolbanovskiy A, Liu Y, Ren J, et al (2007) Sequence context- and temperature-dependent nucleotide excision repair of a benzo[*a*]pyrene diol epoxide-guanine DNA adduct catalyzed by thermophilic UvrABC proteins. Biochemistry 46: 7006–7015. 17506530 10.1021/bi700294k

[44] Christensen LA, Wang H, Van Houten B, Vasquez KM (2008) Efficient processing of TFO-directed psoralen DNA interstrand crosslinks by the UvrABC nuclease. Nucleic Acids Res 36: 7136–7145. 18996898 10.1093/nar/gkn880 PMC2602775

[45] Nakano T, Katafuchi A, Shimizu R, Terato H, Suzuki T, et al (2005) Repair activity of base and nucleotide excision repair enzymes for guanine lesions induced by nitrosative stress. Nucleic Acids Res 33: 2181–2191. 15831791 10.1093/nar/gki513 PMC1079971

[46] Croteau DL, DellaVecchia MJ, Perera L, Van Houten B (2008) Cooperative damage recognition by UvrA and UvrB: identification of UvrA residues that mediate DNA binding. DNA Repair 7: 392–404. 18248777 10.1016/j.dnarep.2007.11.013 PMC2396233

[47] Huang JC, Hsu DS, Kazantsev A, Sancar A (1994) Substrate spectrum of human excinuclease: repair of abasic sites, methylated bases, mismatches, and bulky adducts. Proc Natl Acad Sci U S A 91: 12213–12217. 7991608 10.1073/pnas.91.25.12213 PMC45407

[48] Branum ME, Reardon JT, Sancar A (2001) DNA repair excision nuclease attacks undamaged DNA. A potential source of spontaneous mutations. J Biol Chem 276: 25421–25426. 11353769 10.1074/jbc.M101032200

[49] Moggs JG, Szymkowski DE, Yamada M, Karran P, Wood RD (1997) Differential human nucleotide excision repair of paired and mispaired cisplatin-DNA adducts. Nucleic Acids Res 25: 480–491. 9016585 10.1093/nar/25.3.480 PMC146461

[50] Sugasawa K, Okamoto T, Shimizu Y, Masutani C, Iwai S, et al (2001) A multistep damage recognition mechanism for global genomic nucleotide excision repair. Genes & Dev 15: 507–521. 11238373 10.1101/gad.866301 PMC312644

[51] Mu D, Tursun M, Duckett DR, Drummond JT, Modrich P, et al (1997) Recognition and repair of compound DNA lesions (base damage and mismatch) by human mismatch repair and excision repair systems. Mol Cell Biol 17: 760–769. 9001230 10.1128/mcb.17.2.760 PMC231802

[52] Ohmori H, Friedberg EC, Fuchs RPP, Goodman MF, Hanaoka F, et al (2001) The Y-family of DNA polymerases. Mol Cell 8: 7–8. 11515498 10.1016/s1097-2765(01)00278-7

[53] Sale JE, Lehmann AR, Woodgate R (2012) Y-family DNA polymerases and their role in tolerance of cellular DNA damage. Nat Rev Mol Cell Biol 13: 142–152. 10.1038/nrm3289 PMC3630503 22358330

[54] Yang W, Woodgate R (2007) What a difference a decade makes: insights into translesion DNA synthesis. Proc Natl Acad Sci U S A 104: 15591–15598. 17898175 10.1073/pnas.0704219104 PMC2000391

[55] Albertella MR, Lau A, O'Connor MJ (2005) The overexpression of specialized DNA polymerases in cancer. DNA Repair 4: 583–593. 15811630 10.1016/j.dnarep.2005.01.005

[56] Yang J, Chen Z, Liu Y, Hickey RJ, Malkas LH (2004) Altered DNA polymerase ι expression in breast cancer cells leads to a reduction in DNA replication fidelity and a higher rate of mutagenesis. Cancer Res 64: 5597–5607. 15313897 10.1158/0008-5472.CAN-04-0603

[57] Nick McElhinny SA, Watts BE, Kumar D, Watt DL, Lundstrom EB, et al (2010) Abundant ribonucleotide incorporation into DNA by yeast replicative polymerases. Proc Natl Acad Sci U S A 107: 4949–4954. 20194773 10.1073/pnas.0914857107 PMC2841928

[58] Brown JA, Suo Z (2011) Unlocking the sugar “steric gate” of DNA polymerases. Biochemistry 50: 1135–1142. 21226515 10.1021/bi101915z PMC3040255

[59] Cone R, Duncan J, Hamilton L, Friedberg EC (1977) Partial purification and characterization of a uracil DNA N-glycosidase from *Bacillus subtilis* . Biochemistry 16: 3194–3201. 407925 10.1021/bi00633a024

[60] Lindahl T, Ljungquist S, Siegert W, Nyberg B, Sperens B (1977) DNA N-glycosidases: properties of uracil-DNA glycosidase from *Escherichia coli* . J Biol Chem 252: 3286–3294. 324994

[61] Krokan H, Wittwer CU (1981) Uracil DNA-glycosylase from HeLa cells: general properties, substrate specificity and effect of uracil analogs. Nucleic Acids Res 9: 2599–2613. 7279657 10.1093/nar/9.11.2599 PMC326875

[62] Lujan SA, Williams JS, Clausen AR, Clark AB, Kunkel TA (2013) Ribonucleotides are signals for mismatch repair of leading-strand replication errors. Mol Cell 50: 437–443. 23603118 10.1016/j.molcel.2013.03.017 PMC3658170

[63] Ghodgaonkar MM, Lazzaro F, Olivera-Pimentel M, Artola-Boran M, Cejka P, et al (2013) Ribonucleotides misincorporated into DNA act as strand-discrimination signals in eukaryotic mismatch repair. Mol Cell 50: 323–332. 23603115 10.1016/j.molcel.2013.03.019 PMC3653069

[64] Miller JH (1992) A short course in bacterial genetics: a laboratory manual and handbook for *Escherichia coli* and related bacteria. Cold Spring Harbor, N.Y: Cold Spring Harbor Laboratory Press.

[65] Baba T, Ara T, Hasegawa M, Takai Y, Okumura Y, et al (2006) Construction of *Escherichia coli* K-12 in-frame, single-gene knockout mutants: the Keio collection. Mol Syst Biol 2: 2006.0008. 10.1038/msb4100050 PMC1681482 16738554

[66] Churchward G, Belin D, Nagamine Y (1984) A pSC101-derived plasmid which shows no sequence homology to other commonly used cloning vectors. Gene 31: 165–171. 6098521 10.1016/0378-1119(84)90207-5

[67] Davis BD, Mingioli ES (1950) Mutants of *Escherichia coli* requiring methionine or vitamin B12. J Bacteriol 60: 17–28. 15436457 10.1128/jb.60.1.17-28.1950 PMC385836

[68] Maron DM, Ames BN (1983) Revised methods for the *Salmonella* mutagenicity test. Mutat Res 113: 173–215. 6341825 10.1016/0165-1161(83)90010-9

[69] Garibyan L, Huang T, Kim M, Wolff E, Nguyen A, et al (2003) Use of the *rpoB* gene to determine the specificity of base substitution mutations on the *Escherichia coli* chromosome. DNA Repair 2: 593–608. 12713816 10.1016/s1568-7864(03)00024-7

[70] Wolff E, Kim M, Hu K, Yang H, Miller JH (2004) Polymerases leave fingerprints: analysis of the mutational spectrum in *Escherichia coli rpoB* to assess the role of polymerase IV in spontaneous mutation. J Bacteriol 186: 2900–2905. 15090533 10.1128/JB.186.9.2900-2905.2004 PMC387785

[71] Wang H, DellaVecchia MJ, Skorvaga M, Croteau DL, Erie DA, et al (2006) UvrB domain 4, an autoinhibitory gate for regulation of DNA binding and ATPase activity. J Biol Chem 281: 15227–15237. 16595666 10.1074/jbc.M601476200

