# Supplementary material for: Removal of Misincorporated Ribonucleotides from Prokaryotic Genomes: An Unexpected Role for Nucleotide Excision Repair
Source: PLoS Genet. 2013 Nov 7;9(11):e1003878. doi: 10.1371/journal.pgen.1003878 (PMC3820734; doi:10.1371/journal.pgen.1003878)
Supplement: Figure S1 — In vitro cleavage reactions catalyzed by NER or RNase HII using various DNA-RNA-DNA hybrid templates. The 50-mer duplexes (10 nM) in which the DNA-only strand that is complementary to the rNMP-containing oligonucleotide was 5′ end-labeled (indicated by *), were incubated with either RNase HII, or with the NER proteins and the reaction products were analyzed as described in the legends to Figs. 4 & 5. The sequence of the 50-mer template containing a single rNTP (indicated as “y”) is: 5′-GAC TAC GTA CTG TTA CGG CTC CAT CyA TAC CGC AAT CAG GCC AGA TCT GC-3′. The local sequence context surrounding the ribonucleotide is shown below the gel. The figure demonstrates that the NER and RNase HII mediated incisions observed in Figs. 4 & 5 are specific to the ribonucleotide containing strand, as no incisions are observed on the complementary DNA strand, irrespective of whether it is correctly or incorrectly paired. (PDF) [file pgen.1003878.s001.pdf]

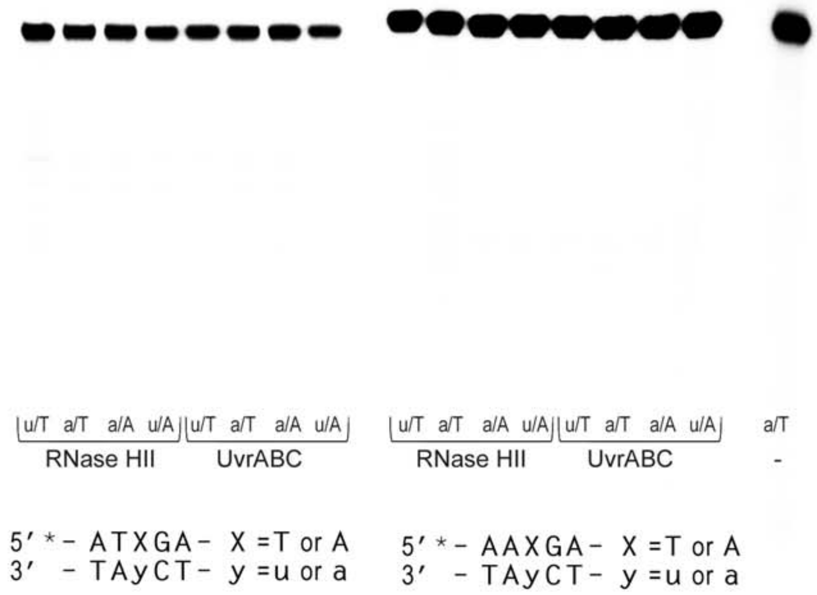
u/T a/T a/A u/A
u/T a/T a/A u/A
u/T a/T a/A u/A
u/T a/T a/A u/A
a/T
RNase HII
UvrABC
RNase HII
UvrABC
-
5′ * - ATXGA - X = T or A
3′ - TAyCT - y = u or a
5′ * - AAXGA - X = T or A
3′ - TAyCT - y = u or a
