# Supplementary material for: Removal of Misincorporated Ribonucleotides from Prokaryotic Genomes: An Unexpected Role for Nucleotide Excision Repair
Source: PLoS Genet. 2013 Nov 7;9(11):e1003878. doi: 10.1371/journal.pgen.1003878 (PMC3820734; doi:10.1371/journal.pgen.1003878)
Supplement: Table S1 — The type of base-pair substitutions generated in the E.coli rpoB gene were determined for recA730 lexA(Def) ΔdinB ΔumuDC strains expressing umuC_Y11A which are proficient- or deficient- in MMR and RNase HII- mediated RER. The data for the rnhB + mutL strain (RW710) are taken from [13] and are shown for direct comparison with the data for the isogenic ΔrnhB mutL strain (RW942). 290–375 RifR mutants were analyzed in the various strain backgrounds. The spectra of mutations obtained in the rnhB + strain represents mutagenic events generated by the DNA polymerase that participates in RER, while the spectra of mutations obtained in the ΔrnhB strains represents persisting mutagenic events in the absence of RER and which are promoted by umuC_Y11A. In both the rnhB + and ΔrnhB strains deficient in MMR, the spectra are dominated by transitions, but the mutagenic hotspots differ depending on the presence, or absence, of RNase HII. Furthermore the ΔrnhB mutL strain exhibits an ∼4-fold increase in the number of transversion mutations that are characteristic of pol V. In the mutL + strains, transitions are efficiently repaired by MMR and as a consequence, the spectra are dominated by transversion events. Similar to the MMR-deficient strains, the location of the mutagenic hotspots depends upon the presence, or absence of RNase HII and are indicative of mutagenic events generated by two different DNA polymerases working in the presence or absence of RER. (PDF) [file pgen.1003878.s002.pdf]

Table S1: Spectrum of spontaneous mutations generated in the *rpoB* gene in a *recA730 lexA*(Def) Δ*dinB* Δ*umuDC mutL*$^{+/-}$ *rnhB*$^{+/-}$ strains in the presence of *umuC*_Y11A[a].

Position[b]	bp change	*rnhB*$^{+}$ *mutL*	Δ*rnhB mutL*	*rnhB*$^{+}$ *mutL*$^{+}$	Δ*rnhB mutL*$^{+}$
1525	AT→CG			2	
1527	CG→AT			2	
1527	CG→GC			26	
1532	AT→CG			2	2
1532	AT→GC	23	11		6
1534	AT→GC	151	85	2	18
1535	CG→TA		2		
1535	CG→TA	88	4	7	
1536	AT→TA			2	
1537	CG→TA	1			
1537	CG→AT			7	
1538	AT→TA		2	72	30
1538	AT→GC	15	41		
1546	CG→AT	1	3	19	22
1546	CG→TA	33	56		6
1547	AT→TA			10	10
1547	AT→GC	11	54	17	10
1576	CG→AT	1	3	7	38
1577	AT→TA	1	20	17	36
1578	CG→AT			5	
1578	CG→GC			24	
1592	CG→AT				6
1592	CG→GC			5	4
1592	CG→TA	17	21	7	
1598	AT→TA			22	4
1600	CG→TA	5			
1691	CG→TA	8	20	2	18
1714	AT→CG			17	12
1714	AT→TA	19	53	7	88
1715	AT→CG			2	
1716	CG→GC			5	
	Transitions	352 (94%)	290 (77%)	36 (12%)	58 (19%)
	Transversions	22 (6%)	85 (23%)	253 (88%)	252 (81%)
	Total	374	375	290	310

[a]: The data are the number of mutants found for each type of base substitution at a particular position
[b]: The numbering system originates from Garibyan *et al*., [57], where the A of the ATG initiation codon is #1.
